# Epigenetic coordination of signaling pathways during the epithelial-mesenchymal transition

**DOI:** 10.1186/1756-8935-6-28

**Published:** 2013-09-02

**Authors:** Marcin Cieślik, Stephen A Hoang, Natalya Baranova, Sanjay Chodaparambil, Manish Kumar, David F Allison, Xiaojiang Xu, J Jacob Wamsley, Lisa Gray, David R Jones, Marty W Mayo, Stefan Bekiranov

**Affiliations:** 1 Department of Biochemistry and Molecular Genetics, University of Virginia, 1340 Jefferson Park Ave, P.O. Box 800733, Charlottesville, VA 22908, USA; 2 Department of Surgery, University of Virginia, Charlottesville, VA 22908, USA

**Keywords:** EMT, Epigenetics, Chromatin, Reprogramming, Feedback

## Abstract

**Background:**

The epithelial-mesenchymal transition (EMT) is a de-differentiation process required for wound healing and development. In tumors of epithelial origin aberrant induction of EMT contributes to cancer progression and metastasis. Studies have begun to implicate epigenetic reprogramming in EMT; however, the relationship between reprogramming and the coordination of cellular processes is largely unexplored. We have previously developed a system to study EMT in a canonical non-small cell lung cancer (NSCLC) model. In this system we have shown that the induction of EMT results in constitutive NF-κB activity. We hypothesized a role for chromatin remodeling in the sustained deregulation of cellular signaling pathways.

**Results:**

We mapped sixteen histone modifications and two variants for epithelial and mesenchymal states. Combinatorial patterns of epigenetic changes were quantified at gene and enhancer loci. We found a distinct chromatin signature among genes in well-established EMT pathways. Strikingly, these genes are only a small minority of those that are differentially expressed. At putative enhancers of genes with the ‘EMT-signature’ we observed highly coordinated epigenetic activation or repression. Furthermore, enhancers that are activated are bound by a set of transcription factors that is distinct from those that bind repressed enhancers. Upregulated genes with the ‘EMT-signature’ are upstream regulators of NF-κB, but are also bound by NF-κB at their promoters and enhancers. These results suggest a chromatin-mediated positive feedback as a likely mechanism for sustained NF-κB activation.

**Conclusions:**

There is highly specific epigenetic regulation at genes and enhancers across several pathways critical to EMT. The sites of these changes in chromatin state implicate several inducible transcription factors with critical roles in EMT (NF-κB, AP-1 and MYC) as targets of this reprogramming. Furthermore, we find evidence that suggests that these transcription factors are in chromatin-mediated transcriptional feedback loops that regulate critical EMT genes. In sum, we establish an important link between chromatin remodeling and shifts in cellular reprogramming.

## Background

Differentiation and lineage commitment occurs through a highly regulated sequence of cellular changes in response to the environment [1]. A conserved de-differentiation process known as the epithelial-mesenchymal transition (EMT) occurs during physiological processes such as development and wound healing [2]. EMT progression involves coordinated cellular remodeling, which results in a less differentiated phenotype in order to reorganize tissue structures. Induction of EMT in epithelial cells results in loss of apical-basal polarity and the adoption of a migratory and invasive mesenchymal phenotype [3]. Recent evidence suggests that inappropriate induction of EMT in tumor cells is associated with the progression of human carcinomas (reviewed in [4,5]). During cancer progression, tumor grade, metastasis, drug resistance, tumor heterogeneity, and cancer stem cell maintenance all correlate with deregulated EMT [6-8].

An increasing body of evidence indicates that the mesenchymal phenotype is established through genome-wide and locus-specific epigenetic reprogramming [9-11]. This suggests that epithelial and mesenchymal phenotypes are coordinated through changes to chromatin states, and a possible role for the so-called ‘histone code’ in EMT [12,13]. According to one hypothesis, phenotypic switches depend on the chromatin-mediated stabilization of transcription factor (TF) activity [14,15]. Although studies have begun to discover mechanistic roles for changes in specific histone modifications during EMT, the combinatorial nature of the reprogramming remains unclear [9].

A number of studies have attempted to discover functional chromatin domains through a computational process referred to as ‘chromatin profiling’ [16,17]. It has been established that combinatorial patterns of posttranslational histone modifications and covalent changes to genomic DNA delineate functional elements within the genome. These histone codes correlate with gene expression and function, enable the *de-novo* discovery of genomic features such as transcription start sites and cis-regulatory regions [17,18], and also aid in specifying cell lineages [19]. As a result, association between chromatin profiles and molecular function has been reported on the basis of GO-term enrichments [16,20-22]. Therefore, we sought to discover patterns of histone modifications that contribute to epigenomic reprogramming during EMT, and how changes in these modifications relate to the signaling events that are known to establish the mesenchymal phenotype.

We clustered chromatin profiles, and discovered that genes and pathways involved in EMT show essentially the same changes in all sixteen histone modifications, and two variants that we profiled. We also see coordinated changes at their local enhancers. Strikingly, these genes represent a small minority of the total set of differentially expressed genes. Our results suggest that specific changes in histone modifications coordinate the regulation of genes and pathways involved in EMT. In concordance with previous research that demonstrates the epigenetic regulation of enhancer activity, we reveal distinct changes in chromatin at enhancers during EMT [23-25]. Furthermore, we show that the directionality of these changes can be distinguished by enrichments for the known binding sites of two different groups of transcriptional regulators. Results from our analyses are all consistent with a model of transcriptional feedback loops mediated by shifts in chromatin states. Our data-driven and integrative computational approach reveals broad epigenetic coordination of transcription factors and signaling cascades with established roles in EMT. We put forward the hypothesis of positive feedback loops involving the NF-κB and AP-1 TF families, and analogous repression of feedback involving MYC.

## Results and discussion

### General strategy

Given the current research that implicates epigenetic mechanisms in the regulation of EMT, we hypothesized that epigenetic reprogramming broadly coordinates cellular processes that contribute to the phenotypic switch. Furthermore, we hypothesized that this coordination occurs in cancer cells that undergo EMT, despite their mutational landscape and genomic instability. Our goal was to discover a shared epigenetic signature between known EMT drivers and further evidence of epigenetic coordination.

**Figure 1:**
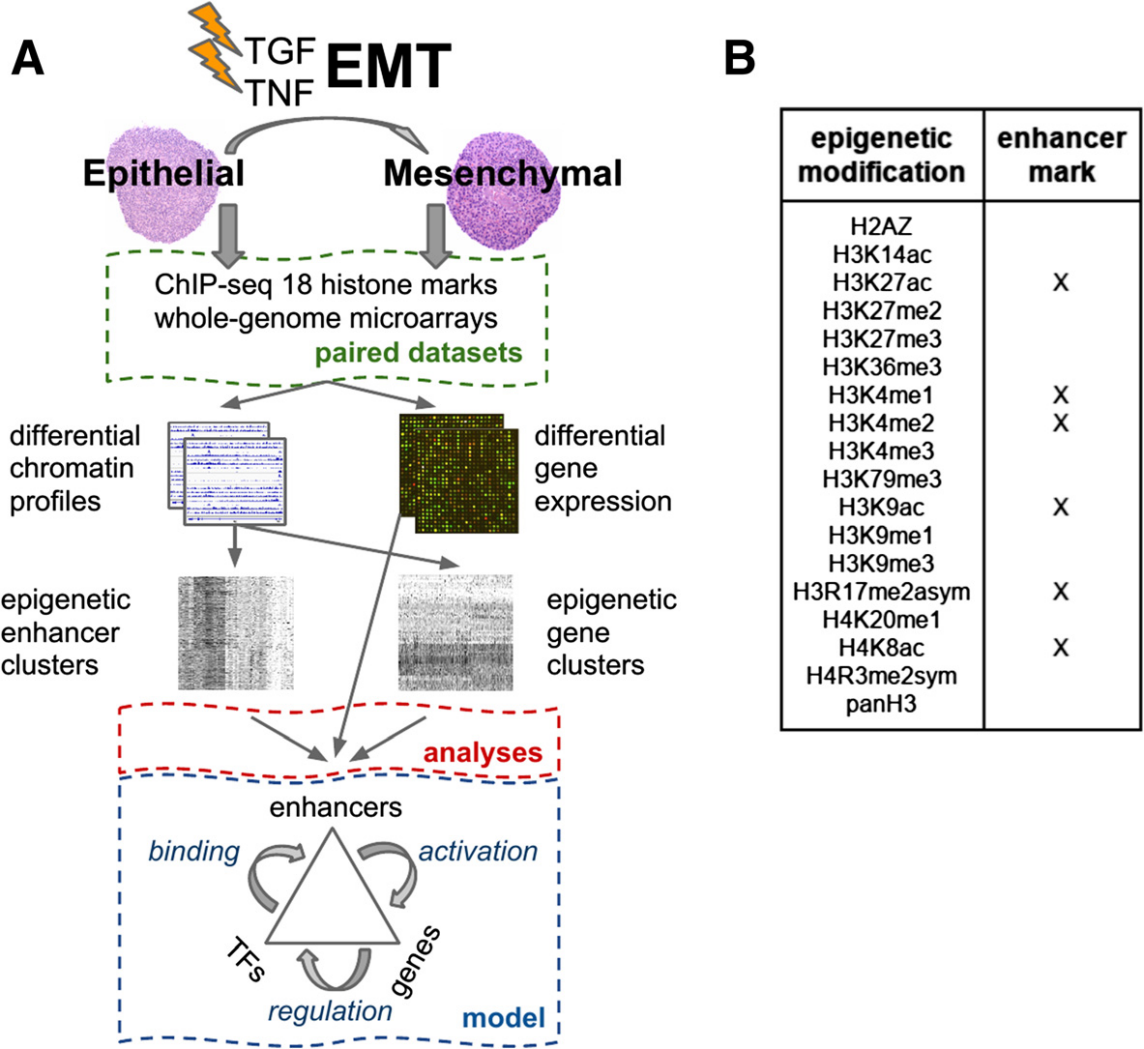
**Experimental design and data. (A)** Flow-chart of the experimental setup and analysis methodology. The epithelial-mesenchymal transition (EMT) was induced using TNF and TGFβ in spheroid cultures. Cells were collected before and after treatment (4 days), and whole-genome gene expression and chromatin profiles of 18 histone modifications and variants were obtained. From the paired data sets we measured differential gene expression and calculated differential epigenetic profiles (DEP). The DEPs were calculated individually for gene and enhancer loci and subsequently clustered. Analyses of the resulting epigenetic gene and enhancer clusters included functional enrichment profiling, network partitioning / ranking, and transcription factor (TF) binding. The results were shown to be consistent with a chromatin-mediated feedback model that involves specific TFs binding activated enhancers that upregulate expression in EMT-related gene clusters. **(B)** Table of histone modifications assayed. Histone modifications shown to be correlated and enriched at enhancer loci are indicated.

To test our hypothesis, we mapped sixteen histone modifications, two histone variants, and collected gene expression data in 3D cultures of untreated (epithelial) and cytokine-treated (mesenchymal) A549 cells (Figure 1A). Briefly, our model system consists of creating three-dimensional NSCLC A549 cultures by hanging droplet [26], and subsequently treating the spheroids with tumor necrosis factor (TNF) and transforming growth factor beta (TGFβ) to induce EMT (Figure 1A). Similar protocols have been utilized to induce EMT in other cell types [27]. This model has been shown to recapitulate critical characteristics of EMT. Reprogrammed cells are shown to have a migratory phenotype, metastatic potential, stem-cell characteristics, and mesenchymal markers. Specifically, we have demonstrated an increase in the expression of master switch EMT transcription factors, TWIST1, SNAI1, SNAI2 and ZEB2, and robust upregulation of stem-cell markers, including KLF4, SOX2, POU5F1/Oct4, MYCN, and KIT. We have also shown loss of CDH1, gain of VIM, greatly increased invasiveness, and increased ability to form lung metastases in nude mice. Importantly, we have demonstrated that, in this particular system, functional characteristics of EMT are dependent on the activity of RELA (p65) (Kumar, M *et al.*, PLOS ONE, in press).

The set of histone marks that were mapped includes those that preferentially associate with transcription start sites, gene bodies, enhancers, or heterochromatin, as well as poorly characterized marks (Figure 1B) [25,28-31]. We and others have shown that many of the mapped marks correlate with transcriptional activity [32]. Here we find a subset of marks correlated at enhancer loci (Figure 1B, [see Additional file 1: Figure S1]). These data were used to quantify the differences in enrichment of each histone modification at gene and enhancer loci. To classify genes (and separately, enhancers) based on their differential epigenetic profiles (DEPs), we employed an unsupervised clustering technique [33]. This effectively groups genes (or enhancers) that share highly similar DEPs across the eighteen chromatin marks analyzed. We then used these gene and enhancer clusters as the foundation of our functional downstream analyses that integrate multiple sources of functional annotations and molecular data (Figure 1A). Specifically, unsupervised clustering enabled us to identify patterns of chromatin remodeling, which we link to signaling pathways and transcription factor activity associated with EMT through comprehensive systems-level analyses.

### Chromatin profiling reveals epithelial-mesenchymal transition-related gene clusters

**Figure 2:**
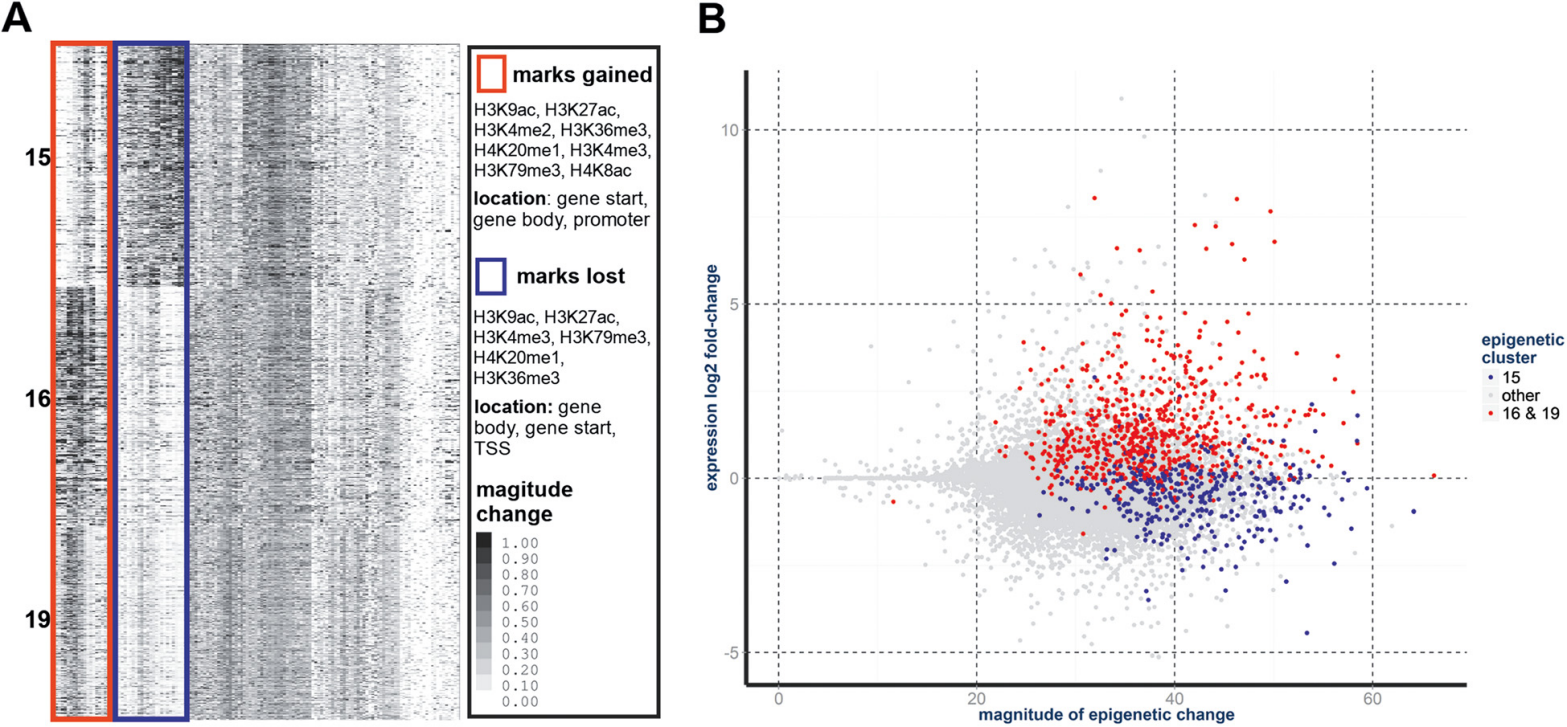
**Epithelial-mesenchymal transition-related gene clusters (EMT-GCs) are differentially expressed and show antipodal patterns of chromatin remodeling. (A)** Differential epigenetic profiles (DEPs) of the EMT-GCs. Heat map shows the DEPs of genes (rows) from the EMT-GCs (other clusters are omitted). Groups of DEP columns that distinguish clusters 16 and 19 from 15 are indicated through colored boxes. Summary of the antipodal patterns of change in histone modification levels are provided in the table. The red box shows changes specific to clusters 16 and 19. The blue box shows changes specific to cluster 15. **(B)** EMT-GCs in the differential expression-epigenetic plane. Each dot represents a gene, colored dots are genes from the EMT-GCs: 16 and 19 (red), and 15 (blue). Differential gene expression (log2 fold-change) is on the Y-axis. The total magnitude of epigenetic difference (sum of DEP elements) at a gene locus is on the X-axis.

Genome-wide application of our clustering methodology with the combined ChIP-seq data yielded twenty-nine non-overlapping gene clusters (GCs). Briefly, our method clusters genes based on the epigenetic profile of gains (positive difference of normalized levels of ChIP-seq enrichment between the mesenchymal and epithelial states) and losses (negative difference) of histone modifications at gene loci during EMT. Each gene locus was partitioned into four segments: promoter, transcription start site (TSS), early gene, and gene body [see Additional file 2: Figure S2]. It should be noted that genes within a given cluster display highly similar profiles of positive and negative differences across the sixteen histone modifications and two variants (Figure 2A). This profile similarity likely occurs because the genes within a cluster undergo similar epigenetic regulation and recognizably distinct regulation of genes from different clusters.

**Figure 3:**
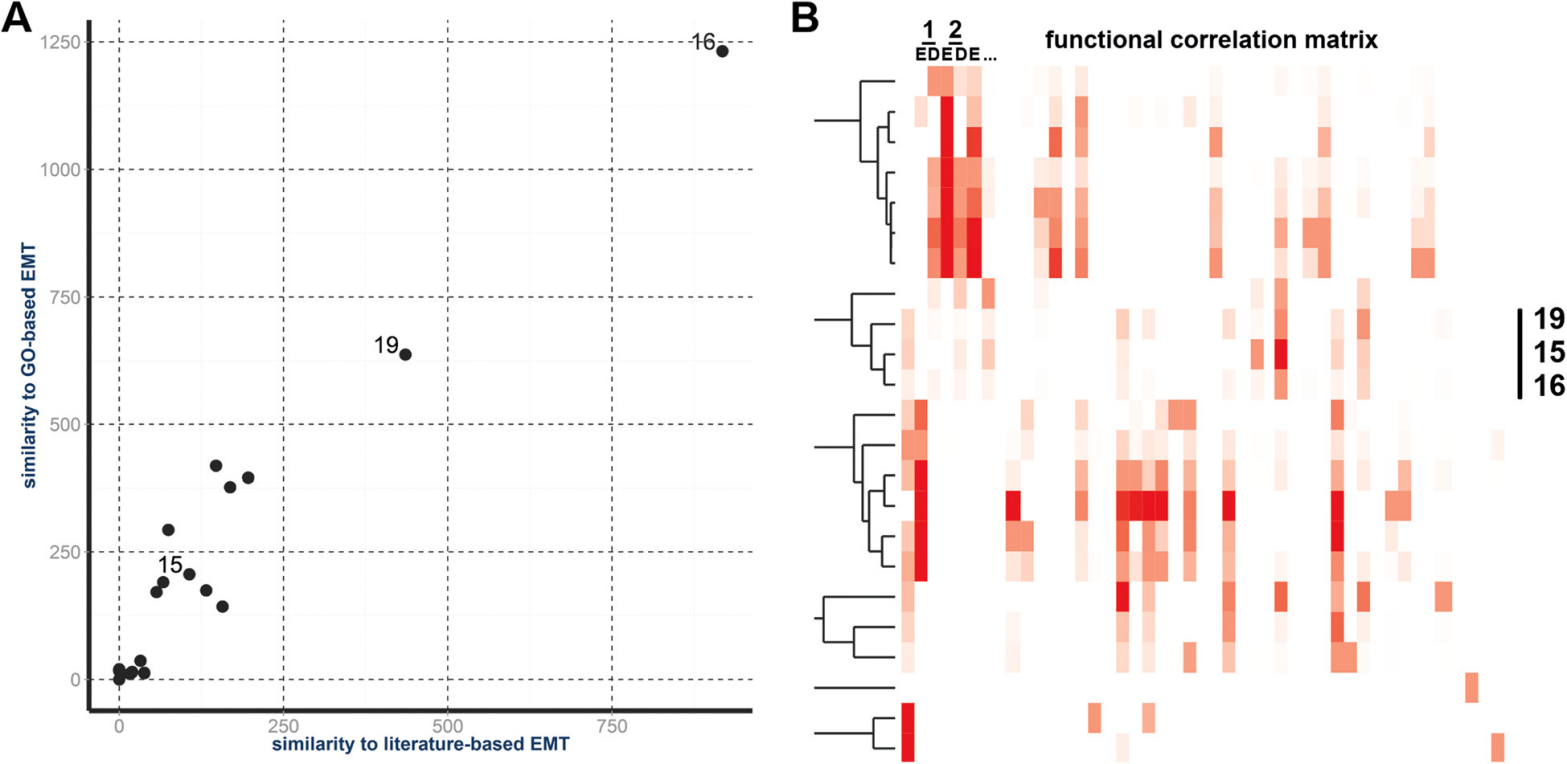
**Epigenetic clustering groups functionally similar genes and identifies epithelial-mesenchymal transition (EMT)-related clusters. (A)** Assessment of EMT functions in gene clusters. Degree of functional similarity between the epigenetic gene clusters and two lists of genes associated with EMT corresponding to genes obtained by manual literature mining and those annotated with GO-terms that included EMT. Functional Similarity Scores (FSS) of each cluster to the two reference EMT gene lists are plotted. **(B)** Functional similarity of gene clusters. Heat map shows the hierarchical clustering of the Functional Correlation Matrix of epigenetic gene clusters. A trimmed dendrogram of the clustering is shown. Each row represents a ‘source’ gene cluster while each column represents either the enrichment (E) or depletion (D) score with a ‘target’ cluster. The sum of the E and D scores is the FSS for a given cluster pair. Columns are arranged numerically by cluster ID.

To identify clusters that are associated with known EMT biology, we looked for enrichments in a subset of GO-derived molecular functions that are enriched among genes known to be involved in EMT. Two clusters, GC16 (378 genes) and GC19 (305 genes) (Figure 2A), are enriched for many of the same GO-terms as a literature-based reference list of EMT-associated genes [see Additional file 3: Table S1] and a similar list of genes annotated with GO-terms explicitly referencing EMT [see Additional file 4: Table S2]. We quantify this degree of overlap and refer to it as functional similarity (Figure 3A, [see Additional file 5: Table S3]). Genes within these clusters have increased expression (Figure 2B), and possess similar patterns of chromatin remodeling (Figure 2A, [see Additional file 6: Figure S3]). We have listed the most significant EMT GO terms for GC16 in Additional file 7: Table S4 (for example, cell adhesion, False Discovery Rate (FDR) corrected *P* value <1e-5). A third cluster, GC15 (385 genes), had a more modest functional similarity to the reference list of EMT-associated genes, but had high functional similarity to GC16 and GC19 (Figure 3B, [see Additional file 5: Table S3]). However in contrast, GC15 shows a global decrease in expression (Figure 2B, [See Additional file 8: Table S5]). The similarity of GC15, GC16, and GC19 in terms of significant GO-terms suggests that genes from these three clusters are engaged in a focused and coordinated process that drives EMT. We refer to these three gene clusters as EMT-related gene clusters (EMT-GCs) and focus our attention on their characteristics and functional similarities (Figures 2 and 3). In subsequent analyses, we provide evidence that EMT is driven by genes in these clusters. Remarkably, the EMT-GCs represent only 5.2% of all 20,707 analyzed genes, compared to 18.5% that are differentially expressed at 5% FDR [See Additional file 8: Table S5]. Compared to differentially expressed genes [see Additional file 7: Table S4; see Additional file 9: Table S6], EMT-GCs show more significant and specific functional enrichments. Thus, analysis of chromatin profiles enabled us to narrow down the search for genes coordinated during reprogramming and enrich for EMT-regulators over differentially expressed passenger genes.

We find, in general terms, that the EMT-GCs are distinguished by relatively large gains (GC16, GC19) and losses (GC15) of activating histone modifications (Figure 2A, Additional file 6: Figure S3). We inspected the patterns of epigenetic remodeling to discover which of the assayed marks most uniquely identify the EMT clusters. We find that in GC15, the histone modifications H4K20me1, H3K79me3, H3K27ac, H3K4me3, and H3K9ac are lost throughout gene bodies. Overall, the epigenetic changes in GC19 are very similar to GC16 with some exceptions. GC16 and GC19 show relatively strong gains of H3K4me2/3, H3K36me3, H4K20me1, H3K9ac, and H3K27ac across gene bodies. Relative to GC16, gains in GC19 are large for H3K79me3, and moderate for H3K27ac, H3K9ac, and H3K4me2/3 in gene bodies. Consistent with their chromatin changes, GC15 and GC16 display the most antipodal changes in gene expression (Figure 2B, [see Additional file 8: Table S5]). By comparison, clusters other than the EMT-GCs exhibit small magnitudes of chromatin and expression changes [see Additional file 6: Figure S3; see Additional file 10: Figure S4]. These observations are in agreement with many findings concerning the broad role of epigenetics in transcriptional regulation and the transcriptional effects associated with specific marks [34-36].

### Epithelial-mesenchymal transition clusters are enriched for many epithelial-mesenchymal transition-associated functions and phenotypes

In order to associate the EMT-GCs with a more comprehensive set of molecular functions and biological processes we profiled them for enrichments for all GO-terms. We removed a large fraction of spurious associations using a 1% FDR cutoff, which revealed that clusters GC16 and GC19 show strong GO enrichment profiles (50 and 23 significant terms, respectively). We found hallmark EMT-regulatory GO-terms, such as cell adhesion and migration, in GC16 and GC19 (Table 1). The terms ‘cell motility’, ‘basement membrane’, ‘stress fiber’, and ‘focal adhesion’ are robustly enriched in GC16 and/or GC19. GO-terms related to the physiological role of EMT such as, ‘wound healing’ and ‘developmental process’ also appeared in these clusters, while GC19 overlaps with the term ‘cell morphogenesis’. In contrast, GC15 has only five significant terms, four of which are associated with development and growth (Table 1). Together, these GO-based analyses reveal a broad similarity of GC15, GC16, and GC19 and association with multiple aspects of EMT, despite differences in the enrichment for specific GO-terms.

**Table 1:** Referenced GO-terms enriched in the epithelial-mesenchymal transition-related gene clusters (EMT-GCs)

**Gene cluster**	**GO-term**	**Enrichment**	***P *value**
16	Wound healing	13.568	0.00001057
16	Plasma membrane	1.982	0.00018160
16	Receptor binding	4.840	0.00024000
16	Seq-spec DNA binding TF activity	2.580	0.00600000
16	Signal transduction	2.523	<1e-8
16	Cellular process	2.651	<1e-8
16	Cell communication	2.358	<1e-8
16	Cell motility	4.231	<1e-8
16	Basement membrane	8.739	0.00959450
16	Cell differentiation	3.078	<1e-8
16	Aging	6.851	0.00000083
16	Growth	3.286	0.00008581
16	Cell death	3.859	<1e-8
16	Cell proliferation	3.901	<1e-8
16	Negative regulation of apoptosis	6.253	0.00000023
16	Immune system process	2.988	<1e-8
16	Cytokine production	4.981	0.00000346
16	Developmental process	3.105	<1e-8
16	MAP kinase tyr/ser/thr phosphat activity	34.020	0.02600000
16	Inactivation of MAPK activity	20.460	0.02400000
16	Pos reg of NF-kappaB TF activity	9.340	0.00150000
19	Plasma membrane	2.022	0.00142517
19	Signal transduction	2.790	<1e-8
19	Cellular process	2.108	0.00001248
19	Cell communication	2.671	<1e-8
19	Cell motility	3.425	0.00023150
19	Focal adhesion	8.441	0.00341880
19	Cell differentiation	2.532	0.00000486
19	Cell death	2.519	0.00059504
19	Cell proliferation	2.765	0.00016907
19	Immune system process	2.302	0.02549018
15	Cellular process	2.010	0.00000250
15	Sequence-specific DNA binding	2.990	0.02700000
15	Developmental process	1.930	0.00042000
15	Cell differentiation	1.910	0.05200000
15	Cell death	2.220	0.00380000
15	Anatomical structure development	1.980	0.00098000
15	Cell proliferation	1.980	0.00690000

GO-terms significantly enriched in GC15, GC16, and GC19. Only GO-terms directly referenced in the manuscript are shown. GO-term annotations are obtained from GOA and NCBI. Enrichment is the fold-change relative to the background frequency of a GO-term annotation. *P* values are calculated by Fisher’s Exact Test and are false discovery rate (FDR) corrected.

Since pathological EMT is linked to metastasis and aggressive tumors, we hypothesized that the genes in the EMT-GCs are associated with advanced cancer phenotypes. To test this hypothesis, we assessed the overlap between these clusters and the sets of genes that distinguish advanced, aggressive cancers from less advanced cancers. These genes sets were obtained from the Molecular Signatures Database 3.0 (MSigDB) [37]. We observe that genes overexpressed in mesenchymal versus luminal types of breast cancer [38] are over-represented in GC16 and GC19 (fold enrichment over background: 9.4, FDR-corrected *P* value: 2.3e-30) and (fold 9.6, *P* 1.3e-25), respectively. Consistently, the downregulated genes from the same study are enriched in GC15 (fold 3.7, *P* 0.0002). Further analysis revealed that GC16 shows significant enrichment for genes upregulated in the peripheral versus the central part of pancreatic tumors (fold 5.4, *P* <1e-5) [39]. This cluster also contains genes that distinguish metastatic tumors from primary colorectal carcinomas (fold 7.89, *P* <1e-5) [40]. In summary, significant overlaps of EMT-GCs with expression signatures of several advanced cancers suggests that tumors of epithelial origin have a common EMT-associated epigenetic mechanism that contributes to progression and metastasis [see Additional file 11: Table S7].

### Regulation of epithelial-mesenchymal transition signaling pathways is chromatin-mediated

Among the GO-terms enriched for GC16 and GC19 are several that correspond to a generic level of many different pathways (for example, ‘receptor binding’, ‘signal transduction’, ‘protein kinase activity’, and 'transcription factor activity' (Table 1 and [see Additional file 7: Table S4]). We hypothesized that chromatin remodeling coordinates the activity of a signaling cascade across all levels of a specific pathway. Since GO-terms only identify functional layers shared by multiple pathways, rather than whole independent pathways, we assessed whether EMT-GCs are enriched for genes from a collection of known pathways. This analysis provides evidence for broad coordination of genes involved in EMT- and cancer-related pathways through chromatin remodeling (pathways referenced in this section are listed in Table 2, [all enriched pathways are in Additional file 12: Table S8]). In addition to several novel insights, we recapitulated many of the pathways and processes that represent the canonical EMT phenotype. For example, both upregulated clusters are enriched for ‘focal adhesion’, ‘ECM-receptor interaction’, ‘adherens junctions,’ ‘tight junctions,’ and E-Cadherin (*CDH1*) related pathways. GC19 shows enrichment for additional pathways involved in cell motility such as ‘regulation of actin cytoskeleton,’ and ‘leukocyte transendothelial migration’.

**Table 2:** Referenced pathways enriched in the epithelial-mesenchymal transition-related gene clusters (EMT-GCs)

**GC**	**Pathway name**	**Enrichment**	**p-value**
16	Pathways in cancer	4.618	0.00000627
16	Direct p53 effectors	8.279	0.00000023
16	p53 signaling pathway	7.100	0.09600000
16	Focal adhesion	5.298	0.00013609
16	ECM-receptor interaction	6.963	0.01242671
16	Cytokines and inflammatory response	18.189	0.00875280
16	Interleukin-1 processing	54.274	0.01740033
16	T cell receptor signaling pathway	8.320	0.00000663
16	TNF-alpha/NF-kB signaling pathway	4.280	0.03567735
16	CD40/CD40L signaling	13.097	0.04278382
16	MAPK signaling pathway	3.493	0.09603616
19	Pathways in cancer	5.303	0.00000226
19	Focal adhesion	6.245	0.00003282
19	E-cad sig in the nasc adherens junction	24.776	0.00000267
19	Regulation of actin cytoskeleton	6.012	0.00571942
19	Adherens junction	13.011	0.00000273
19	Junction	14.070	0.00496435
19	Canonical NF-kappaB pathway	20.435	0.00422071
19	MAPK signaling pathway	4.918	0.08357500
19	Leukocyte transendothelial migration	8.442	0.00006173
19	T cell receptor signaling pathway	8.321	0.00000663
19	TGF-beta receptor signaling	15.678	0.00001359

Pathways significantly enriched in GC16 and GC19. Only pathways directly referenced in the manuscript are shown. Pathways have been sourced from the NCBI Biosystems. Enrichment is the fold-change relative to the background frequency of a pathway annotation. *P* values are calculated by Fisher’s Exact Test and are false discovery rate (FDR) corrected.

Since we assessed the histone modification and expression levels from cells that had been exposed to TNF and TGFβ over an extended time course, we expected to find delayed early and late response genes within the EMT-GCs. Some well known delayed early and late genes confirmed our hypothesis, including *EGFR* (GC16, log2 fold-change: 2.45), *SNAI2* (GC16, log2fc 4.06), *INHBA* (GC16, log2fc 8.01), *INHBB* (GC15, log2fc −3.24), *COL1A1* (GC16, log2fc 4.25), *SKIL* (GC19, log2fc 3.22), *TGFBR1* (GC19, log2fc 3.53). Surprisingly, we also observed persistent epigenetic and transcriptional activation of genes associated with the immediate early response to TNF and TGFβ exposure. Gene expression profiling indicates that many immediate early genes (IEGs) remained upregulated rather than returning to basal levels. For example *JUN*, *MAF*, *MYCN*, and *KLF7* show strong overexpression and have an active chromatin profile (GC16 and GC19). Other IEGs including *JUNB*, *GADD45B*, *ZFP36*, *ZFP36L1*, *HES1*, *EPHA2*, *IER3*, *SOX9*, and *MAFG* show moderate overexpression, but appear in the epigenetically repressed GC15. In many cases, IEGs are induced by MAP kinase (MAPK) signaling after growth hormone stimulation [41]. These IEGs then induce the transcription of delayed early genes (DEGs). A negative feedback mechanism exists through the repressive activity of DEGs on IEG expression and MAPK signaling.

We observed that the EMT-induced cells upregulated protein phosphatases that attenuate MAPK signaling, including dual-specificity phosphatases (DUSPs). The EMT-GCs contained a significant number of these phosphatases. Specifically, GC16 and GC19 contain *DUSP1*/*5*/*6*/*8*/*10*/*16*, while *DUSP4* is a member of GC15. We gained additional support for the activation of MAPK attenuation through GO analysis. We found that GO-terms for ‘MAP kinase phosphatase activity’ and ‘inactivation of MAPK activity’ were enriched in GC16 (Table 1). In summary, we observed sustained IEG expression despite an enrichment of DUSP family members in the EMT clusters. The apparent continued transcription of both IEGs and DUSPs, well beyond the early response, suggests loss of negative feedback regulation of MAPK signaling in our system.

We used TNF as a proinflammatory cytokine to enhance TGFβ-induced EMT in our model system, and we find that genes that propagate TNF signaling are upregulated and strongly enriched in GC16 and GC19. Specifically, the TNF / NF-κB signaling pathway is enriched in both upregulated EMT-GCs, while GC16 is enriched for signaling from the TNF receptor, CD40. An enrichment of genes related to the ‘positive regulation of NF-κB’ in GC16 further supports sustained NF-κB activity. Interestingly, cluster GC15 also contains several NF-κB-related proteins. For example, we observed downregulation of the β-arrestin 1 and 2 genes (*ARRB1*/*2*, log2fc −1.62 and −2.61, respectively). Arrestins show increased expression in differentiated cells and inhibit cellular responses to growth stimuli. Although, their role in EMT remains unclear, overexpression of either *ARRB1* or *ARRB2* in HeLa cells inhibits NF-κB-mediated transcription. This inhibition occurs primarily through interactions and stabilization of IκBα (*NFBIA*), as well as interactions with the IκB kinases [42,43]. Clinical data shows that serum levels of arrestins are lower in patients with NSCLC, and that these decreased levels correlate with poor survival [44]. In our system we have validated that constitutive activity of NF-κB is required for induction of EMT and potentiates a mesenchymal phenotype (Kumar, M *et al.*, PLOS ONE, in press). Taken together, these data indicate that constitutive NF-κB activation during EMT occurs through the epigenetic reprogramming of genes that regulate TNF signaling.

The EMT-GCs also contain many genes that participate in the EGFR signaling pathway, including the receptors themselves. The *EGFR* gene is upregulated and contained in GC16, while *ERBB2* and *ERBB3* (GC15) are significantly downregulated (log2fc −2.30 and −2.04, respectively). Upregulation of the active ErbB2/3 heterodimer occurs in more differentiated cancers, and therefore downregulation of *ERBB2*/*3* and upregulation of *EGFR* may constitute a receptor switch associated with the core basal phenotype [45]. Such events may affect ligand specificity and enable cellular reprogramming. Importantly, EMT is associated with resistance to EGFR inhibition [46]. This analysis indicates that epigenetic reprogramming contributes to altered EGF signaling in our model system.

Further examination of GC16 and GC19 revealed enrichment for additional pathways broadly associated with cancer and EMT [see Additional file 12: Table S8], most of which overlap or crosstalk with TNF, MAPK, or EGFR signaling. For example, GC16 and GC19 are enriched for genes from large cancer-related pathways including: ‘KEGG: pathways in cancer’, ‘direct p53 effectors’ and the ‘p53 signaling pathway’. Furthermore, the intersection of these pathways includes many highly upregulated genes from the EMT-GCs such as *SNAI2* (log2fc 4.06), *PRDM1* (log2fc 3.60), *JUN* (log2fc 3.62), and *EGFR* (log2fc 2.45). We also observed an overrepresentation of several immune response pathways in the EMT-GCs. GC16 is enriched for the ‘cytokines and inflammatory response’ and ‘interleukin-1 processing’ pathways, while GC19 is enriched for ‘T cell receptor signaling’. These findings agree with recent studies that establish a strong association of paracrine cytokine signaling and inflammatory pathways with EMT and metastatic cancer-progression [47-49].

### Epigenetic switches at enhancers correlate with differential gene expression

Since previous studies have indicated a strong association between the chromatin state at enhancers and expression of proximal genes [31,50-52] we extended our epigenetic analysis to putative enhancer loci. This analysis provided insight into the role of specific TFs in the induction of EMT. Moreover, integration of the gene and enhancer clustering showed coordinated changes in chromatin states at genes and enhancers during EMT.

We hypothesized that differential gene expression correlates with epigenetic modulation of proximal enhancers. To test this hypothesis, we identified 75,937 putative enhancers in epithelial and mesenchymal cells based on promoter-distal H3K4me1 and H3K27ac peaks, which mark enhancers in promoter-distal regions [25]. Next we identified additional ‘enhancer-associated’ marks, which correlate with either H3K4me1 or H3K27ac at these putative enhancer sites [see Additional file 1: Figure S1]. The enhancer-associated marks include H3K4me1/2, H3K27ac, H3K9ac, H4K8ac, and H3R17me2asym. Of the 75,937 putative enhancers, 30,681 were found to be differentially marked by the enhancer-associated marks between the epithelial and mesenchymal states. We then grouped these differential enhancers into thirty-eight clusters based on their differential levels of the enhancer-associated marks. We observed that within a given cluster all enhancer marks had the same trend of either gain or loss. Correspondingly, few clusters show simultaneous gain and loss of different marks. These observations guided our binary division of enhancer clusters into two groups: ‘gain’ or ‘loss’. Within these two broad classes, clusters show distinct magnitudes of change for specific marks [see Additional file 13: Figure S5].

**Figure 4:**
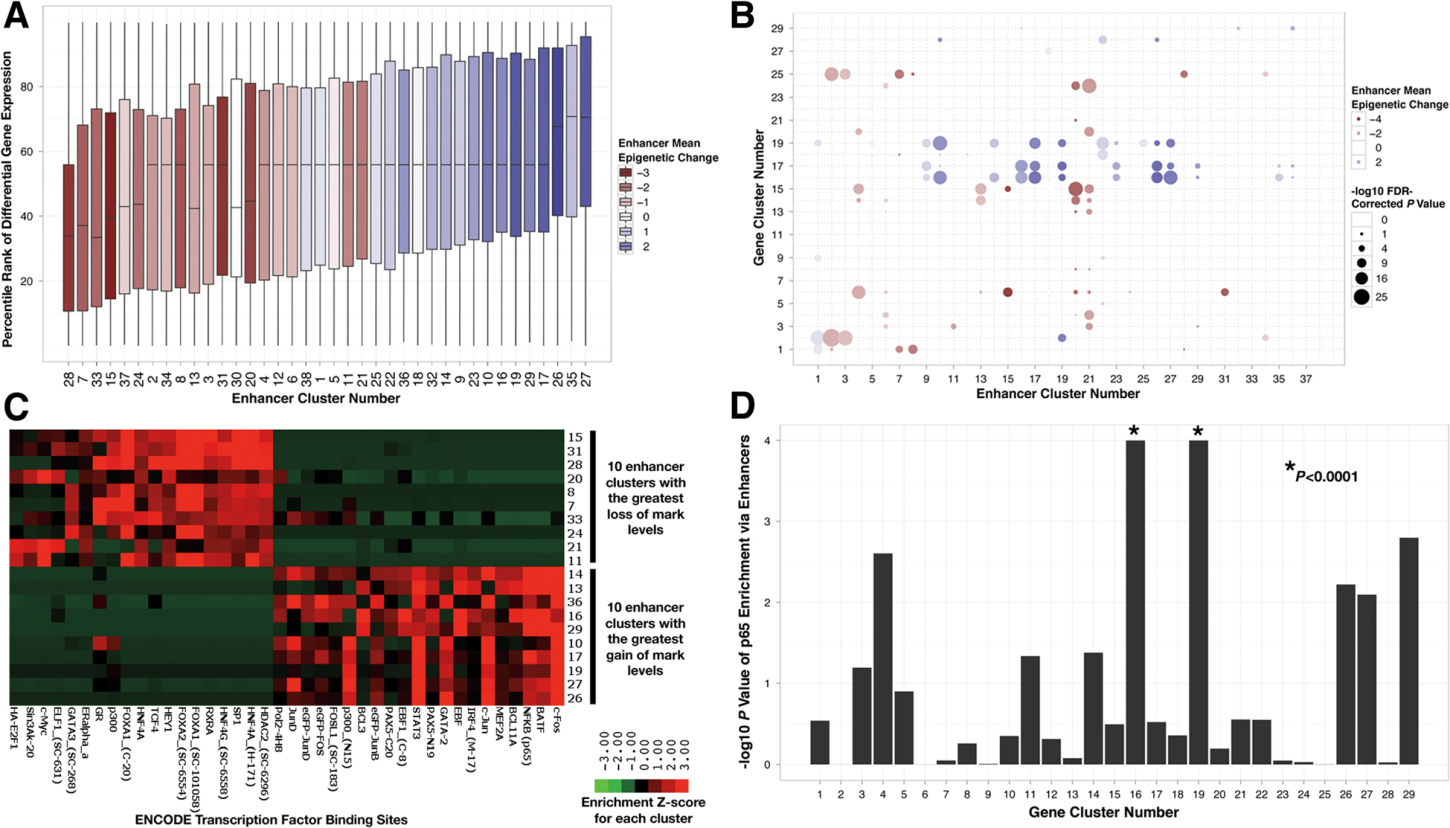
**Activated and repressed enhancers associated with epithelial-mesenchymal transition-related gene clusters (EMT-GCs) and different sets of transcription factors. (A)** Box plots of percentile ranks of differential expression for genes associated with each enhancer cluster. Boxes are colored by average magnitude of gain (blue) or loss (red) of enhancer-associated marks. **(B)** Overlap between gene clusters and genes linked to enhancer clusters. Bubbles are colored with respect to enhancers in the same manner as the boxes in panel A. Size of the bubbles represents the -log10 *P* value of the overlap. **(C)** Association of activated and repressed enhancer clusters with transcription factor binding sites. Significance of overlap between ENCODE transcription factor binding sites (columns) and the 10 enhancer clusters with the strongest activated signatures as well as the 10 equivalent repressed enhancer clusters (rows). Each spot on the heat map is the -log10 *P* value of the overlap, which is Z-score normalized by row. **(D)** Association of p65 binding sites with gene clusters via enhancers. Enrichment of p65 binding sites (ENCODE) in the enhancers assigned to each gene cluster.

The enhancer-associated marks are generally associated with open chromatin and active enhancers, which suggests that gain and loss clusters correspond to activation and repression, respectively. To test the association of enhancer remodeling to gene expression, we assigned a ‘gain-loss’ score to each enhancer cluster. We define this score as the mean of the difference between gains and losses across the enhancer-associated marks. These gain-loss scores of enhancer clusters are strongly correlated with the mean differential expression of genes associated with the clusters (r = 0.89, Figure 4A and [see Additional file 14: Figure S6]). Therefore, our analysis establishes a link between gain clusters and activated genes, as well as a link between loss clusters and repressed genes.

The EMT clusters also showed strong association with differential enhancers relative to other gene clusters (Figure 4B). Examination of these clusters revealed that GC16 and GC19 show striking enrichment for genes associated with activated enhancer clusters. Consistently, GC15 shows strong association with erased enhancer clusters. Interestingly, GC17 also shows overlap with activated enhancer clusters despite lacking noteworthy EMT functional similarity. However, this cluster contains some highly upregulated genes associated with EMT, such as *MMP1*, *MMP9*, and *MMP10*, which are upregulated 453-fold, 278-fold, and 1,910-fold, respectively. Together, these observations indicate a widespread co-regulation of enhancers and genes involved in EMT through chromatin remodeling.

### Transcriptional control of epithelial-mesenchymal transition-related gene clusters through epigenetic reprogramming of enhancers

Because modification of histone tails in enhancer regions influences DNA accessibility, we wanted to determine if the binary regulation (activation or repression) of enhancers corresponds to the binding of specific TFs during EMT. We compared the activated and repressed enhancer clusters for differences in preferential binding of specific TFs. Transcription factors mapped by ENCODE were clustered by the enrichment of their binding sites in enhancer clusters with the lowest and highest gain-loss scores. As expected, the TFs sharply partition into two non-overlapping sets that correspond to enhancer activation and repression (Figure 4C). The presence of this sharp distinction between activated and repressed enhancers indicates that the epigenetic regulation of enhancers is tightly coupled to TF binding.

Several TFs downstream of the pathways enriched in the EMT-GCs (that is, TGFβ, TNF, and EGFR) are enriched in activated and repressed enhancer clusters. For example, p65 (*RELA*), c-Fos (*FOS*), and c-Jun (*JUN*) binding sites show significant enrichment in the activated enhancer clusters. Interestingly, in addition to c-Fos and c-Jun, many AP-1 family members are enriched in the activated enhancer clusters as well, namely fra-1 (*FOSL1*), jun-B (*JUNB*), jun-D (*JUND*), and B-ATF (*BATF*). Together with our pathway analyses, these results demonstrate a chromatin-mediated activation of enhancers that bind NF-κB and AP-1 family members.

We used ENCODE transcription factor binding site data to determine whether NF-κB and AP-1 binding sites associated with the EMT-GCs via binding sites at enhancers. We found a strong association of the p65 binding sites with enhancers linked to GC16 (*P* <0.0001) and GC19 (*P* <0.0001), but a weak association with GC15-linked enhancers (*P* 0.32) (Figure 4D). Moreover, we observed a similar pattern for AP-1 family member binding sites [see Additional file 15: Figure S7]. These results strongly suggest that genes in GC16 and GC19 are regulated through the differential epigenetic activation of enhancers that contain p65 and AP-1 family member binding sites.

**Figure 5:**
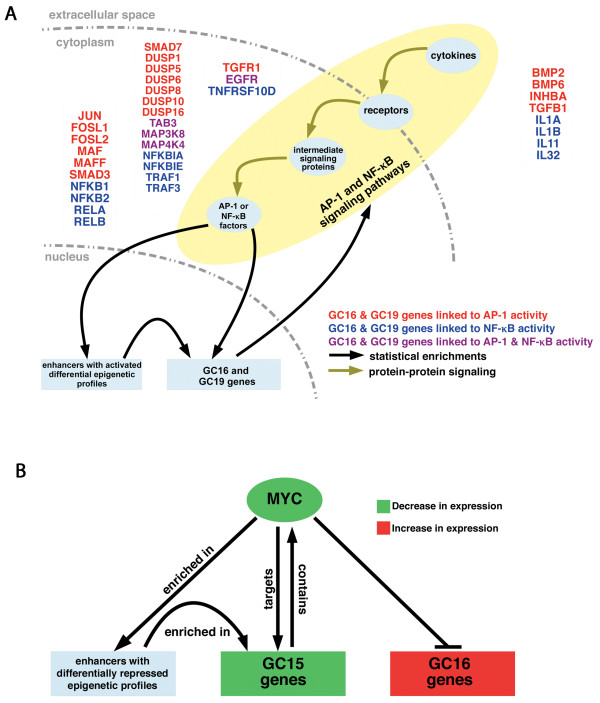
**Evidence for broad feedback regulation by AP-1 and NF-κB family members, and c-Myc. (A)** Statistical enrichments of AP-1 and NF-κB binding sites link these transcription factors to activated enhancers and the upregulated epithelial-mesenchymal transition-related gene clusters (EMT-GCs). EMT clusters themselves are enriched for in pathways and functions associated with positive regulation of AP-1 and NF-κB. Some genes in GC16 and GC19 that are known to regulate either AP-1 or NF-κB are listed. **(B)** c-Myc binding sites are enriched in repressed enhancers and the repressed EMT gene cluster, GC15. Moreover, GC16 is enriched for genes that are repressed by c-Myc.

In addition to the connection between EMT-GCs and activated enhancers that bind AP-1 or NF-κB TFs, we observed other evidence that regulation of these transcription factors contribute to EMT (statistical associations shown in Figure 5A as black arrows). First, AP-1 and NF-κB family members show high transcriptional upregulation, and are found in GC16 and GC19 see Additional file 8: Table S5]. Additionally, genes with predicted AP-1 or NF-κB binding sites in their promoters are enriched in GC16 (fold 5.6, *P* 0.00004) and GC19 (fold 8.9, *P* <1e-5), respectively. GC19 is also enriched for genes with predicted AP-1 binding sites in their promoters (fold 2.7, *P* 0.009). Examination of GC16 revealed a strong enrichment of genes induced by NF-κB signaling in primary human keratinocytes (fold 19.5, *P* <1e-5) and fibroblasts (fold 13.4, *P* <1e-5) [53], as well as the core NF-κB signaling proteins (fold 54.4, *P* 0.003) [54] themselves. Taken together, these results provide evidence that AP-1 and NF-κB are major regulators of the genes in the upregulated EMT clusters (Figure 5A).

Examination of the erased enhancer clusters identified c-Myc as the only enriched TF that is downstream of the pathways enriched in the EMT-GCs. Association of c-Myc binding sites to EMT-GCs via enhancers revealed a significant association with GC15, and a lack of association with GC16 and GC19. It should be noted that this analysis also demonstrates an association between enhancers with c-Myc binding sites and other gene clusters with more modest differential expression [see Additional file 15: Figure S7]. This may be explained by the expansive role of c-Myc in gene regulation [55]. Comparison to experimental data revealed that GC15 possesses significant enrichment for validated c-Myc targets from two sources (fold 4.5, *P* 0.002) and (fold 2.2, *P* 0.04), respectively [56,57]. Furthermore, GC16 significantly overlaps the subset of negatively regulated c-Myc targets [57] (fold 5.7, *P* 7.8e-7), suggesting that c-Myc has opposing transcriptional effects on GC15 and GC16. Finally, from microarray we observed a nearly 2-fold decrease in *MYC* expression after induction of EMT in our system. We validated that *MYC* was in fact downregulated by QT-PCR and observed a significant and almost four-fold reduction in transcript [see Additional file 16: Figure S8]. These results suggest that decreased c-Myc activity contributes to EMT progression in our model system, through both the de-activation and de-repression of genes in the EMT-GCs (Figure 5B).

### Links between enhancer clusters, gene clusters, and transcription factors indicate a mechanism of chromatin-mediated transcriptional feedback

Strikingly, AP-1 and NF-κB transcription factors, as well as c-Myc, reside in the EMT-GCs. Thus, these TFs potentially regulate their own expression and undergo chromatin regulation that is similar to their targets. For example, a large fraction of the AP-1 family of genes reside in the EMT-GCs, including FOSL1 (log2fc 3.12), FOSL2 (log2fc 0.88), JUN (log2fc 3.62), MAF (log2fc 7.27), and MAFF (log2fc 1.21), which are in GC16; while FOS (no significant change), MAFG (log2fc 1.05), JUND (no significant change), and JUNB (log2fc 1.80) belong to GC15. Genes that encode TFs that are not AP-1 family members, but which can heterodimerize with AP-1 members also reside in the EMT-GCs, including CEBPD (GC15, log2fc −3.49), CEBPB (GC15, log2fc 0.89), and CEBPG (GC16, log2fc 0.61). Additionally, GC16 contains three NF-κB family members: NFKB2 (log2fc 1.76), RELA (log2fc 1.23), RELB (log2fc 2.27); NFKB1 (log2fc 1.89) appears in GC19. As expected, the downregulated *MYC* gene resides in GC15. Based on these coordinated changes in chromatin state for a small set of TFs and their respective pathways, enhancer binding sites, and downstream targets, we put forward a hypothetical model that EMT is maintained by chromatin-mediated transcriptional feedback mechanisms involving the TF families that we have highlighted. This model provides a plausible explanation for the sustained activity and critical role of NF-κB in our experimental system.

### Chromatin remodeling coordinates a modular protein interaction network

**Figure 6:**
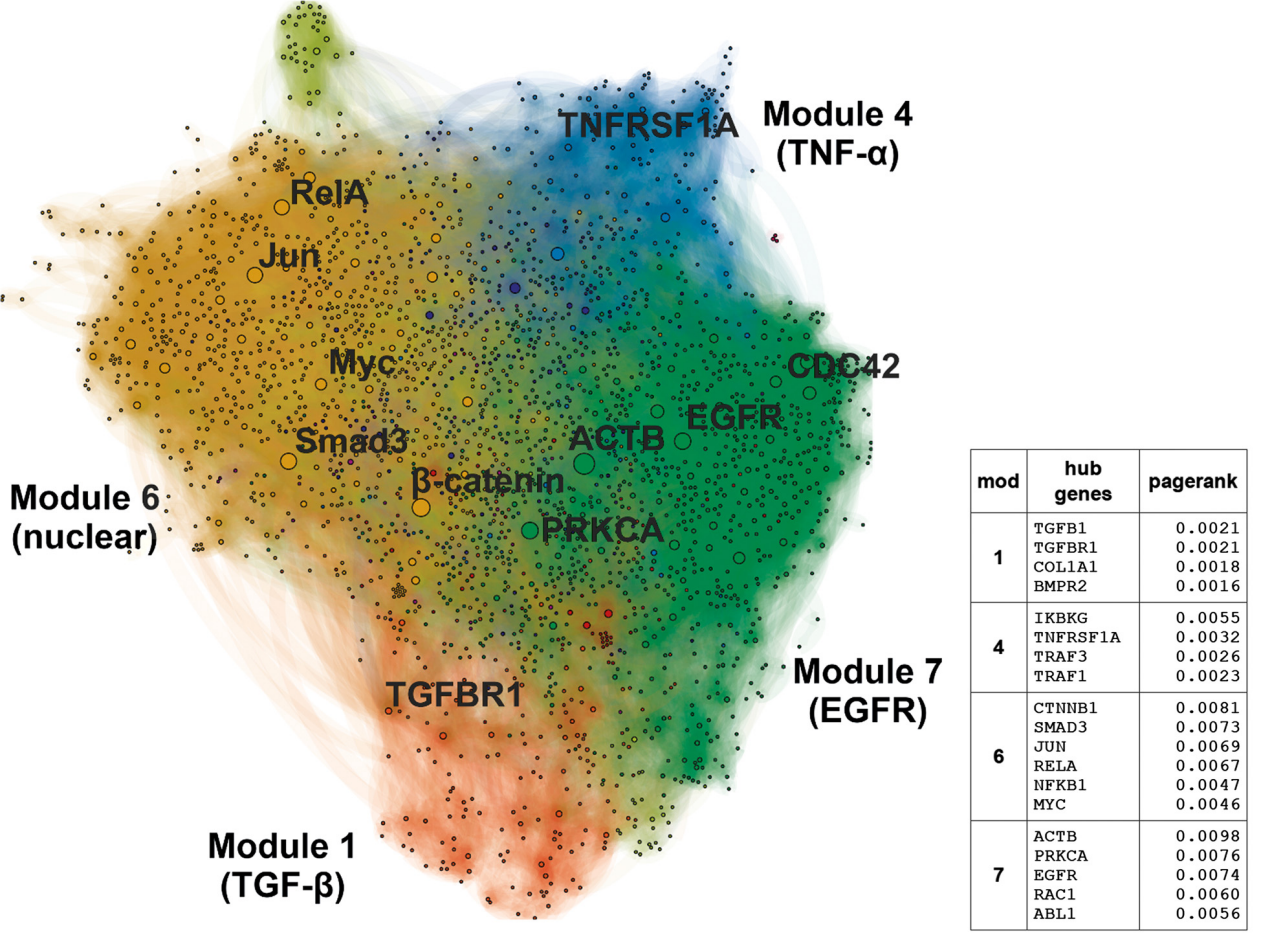
**The translational readout of the epithelial-mesenchymal transition-related gene clusters (EMT-GCs) forms a modular protein-protein interaction network of EMT signaling (EMT-network).** The EMT-network is based on experimentally determined interactions and induced by genes from the EMT-GCs. Nodes are genes from the EMT-GCs and additional genes that directly mediate their interactions. Node sizes are proportional to PageRank scores. Genes with high scores are network hubs. The EMT-network is partitioned into eight modules, four of them labeled and color-coded. The predominant functional characteristic of each module is indicated. The side table lists genes with the highest PageRanks in each of the four core modules.

To understand at the system level how chromatin remodeling coordinates signaling pathways in EMT, we analyzed the gene clusters through an unbiased protein-protein interaction (PPI) network. First, a sub-network (‘EMT-network’, Figure 6) of the whole cell interactome was defined, based on the genes found in the EMT-GCs. We then characterized the network in terms of functions and pathways. The resulting analysis showed integration of several signaling pathways, such as TGFβ, EGF, and TNF, which converge on the TFs that were identified in the enhancer analysis, including AP-1 and NF-κB family members.

We defined the EMT-network as the PPI network that includes all of the genes in the EMT-GCs that connect to each other either directly, or through an intermediate gene, in which case the intermediate gene is included in the network. Therefore, we created a PPI network of genes that show coordinated, EMT-specific chromatin remodeling, along with common immediate neighbors. The EMT-network contains a total of 2,534 genes and 16,922 interactions.

We further resolved the network by delineating ‘hubs’ and ‘modules’. Modules are sets of densely connected genes within a network, and typically contain genes that are functionally associated. By definition, any two modules must show relative independence from each other in terms of connectivity. Hubs are important genes within a network. They mediate interactions among other less connected genes, and determine the modular organization of PPIs [58]. We used the PageRank score to identify hubs, and we used an unsupervised algorithm to delineate the modules [59].

We ranked genes in the EMT-network based on their PageRank (PR). Hubs with the highest PR come exclusively from the EMT-GCs, and include: *ACTB* (rank 1), *CTNNB1* (2), *PRKCA* (3), *EGFR* (4), *RAC1* (8), *ABL1* (9), and a number of TFs: *SMAD3* (5), *JUN* (6), *RELA* (7), and *MYC* (14) [see Additional file 17: Table S9]. By definition these genes are the most important mediators of interactions between genes from EMT clusters and potentially coordinate their function.

We found that the pathways most significantly associated with the network hubs are: (1) the pro-inflammatory TNF signaling cascade through CD40 (fold 2.09, *P* <1e-5) and the canonical NF-κB pathway (fold 2.03, *P* 0.0013), (2) EGF receptor signaling pathways including EGFR (fold 2.01, *P* 0.00090), and ErbB2/3 (fold 2.01, *P* 0.00074), and (3) the TGFβ (fold 1.99, *P* 0.00082) and Wnt (fold 1.92, *P* 0.006) signaling pathways. The enrichment of the hub genes for these pathways, along with their transcriptional regulation, strongly suggests that chromatin maintains the upregulation of these pathways in an EMT-specific manner, hence, driving cells to the mesenchymal state.

### Cytosolic modules within the epithelial-mesenchymal transition-network correspond to distinct signaling cascades

We partitioned the EMT-network into nine modules and focused our analyses on the four largest and most densely connected modules (M1, M4, M6, M7). They contain 86% of the 2,543 genes in the EMT-network, while the remaining six modules were either small or dispersed throughout the network [see Additional file 8: Table S5]. An enrichment of cell surface receptors and membrane proteins exists within three of the modules (M1, M4, M7). We refer to this group as the ‘upstream’ modules. Based on this observation, we hypothesized that distinct network modules could have distinct molecular characteristics. To test this we further characterized the modules through GO-terms, molecular signatures, and pathways. We found that the three upstream modules correspond to three signaling cascades: TGFβ, TNF / NF-κB, and receptor tyrosine kinases.

#### TGFβ receptor signaling

Module M1 most significantly associates with the TGFβ, and BMP signaling pathways, but is also enriched for genes related to development, cell proliferation, apoptosis, and differentiation. From GO, the most enriched biological processes are EMT (fold 35.44, *P* 0.000085) and mesenchymal differentiation (fold 99.73, *P* 0.0080). In terms of pathways, we found that this module is most significantly enriched for the TGFβ pathway (fold 46.20, *P* <1e-5) and other molecular functions related to TGFβ signaling. For example, BMP signaling events (fold 57.47, *P* <1e-5) and proteins known to bind activin A (fold 203.15, *P* <1e-5) are strongly enriched. Both BMPs, and activin A belong to the TGFβ superfamily. Canonically, TGFβ utilizes receptor S/T kinases to activate the SMAD proteins. As expected, we observed overrepresentation of genes that regulate SMADs through phosphorylation (fold 310.28, *P* <1e-5) and mediate their nuclear import (fold 201.15, *P* <1e-5) in M1. These findings indicate that module M1 captures the TGFβ and BMP signaling pathways, which are critical to EMT induction.

#### TNF/NF-κB signaling

Module M4 includes the TNF / NF-κB signaling network and is also enriched for genes from the MAPK signaling pathway. The majority of genes that are annotated as mediators of apoptosis signaling reside in this module. Specifically, M4 contains all annotated genes of the extrinsic apoptosis pathway (*P* <1e-5), and high enrichments for the intrinsic (fold 73.7, *P* <1e-5), general (fold 92.12, *P* <1e-5), and caspase (fold 52.54, *P* <1e-5) apoptosis pathways. Another defining characteristic of M4 is TNF signaling, since all annotated genes in this pathway are included (*P* <1e-5). Consistently, this module contains genes involved in signaling pathways upstream of NF-κB (fold 82.80, *P* <1e-5). Furthermore, we observed enrichment of the IL1 (fold 409.41, *P* <1e-5), Toll-like (fold 29.48, *P* <1e-5), and NOD-like (fold 27.84, *P* <1e-5) pathways. All of these receptors are activated by pro-inflammatory signals, and converge on NF-κB. We also noted an overrepresentation of cytosolic mediators of immune responses. In particular, there are enrichments for the IKK complex (fold 215.98, *P* <1e-5), the TAK1/JNK cascade (fold 104.81, *P* <1e-5), and the MAPK stress activated cascade (fold 19.50, *P* <1e-5). These findings are consistent with the critical role of inflammation in EMT (reviewed in [60]). For example, IL-1 activity is known to induce the *ZEB1* and *ZEB2* master-switch EMT TFs through NF-κB [61]. Furthermore, both TNF and IL-1 induce the expression and nuclear localization of several AP-1 family members, such as *FOSL1* and *FOSB*, in addition to NF-κB [62]. These results suggest, that unlike the developmental and mesenchymal bias in M1, this module associates more strongly with the immune response and apoptosis and groups the interactions important for the propagation of TNF / NF-κB signaling in our model of EMT.

Module M7 includes signaling pathways from cell surface interactions and from receptor tyrosine kinases (RTKs). Cytosolic and signal transduction proteins show significant enrichment in this module (fold 5.07, *P* 1.25e-86; and fold 4.86, *P* 4.0e-55, respectively). We found several EGF receptor signaling pathways overrepresented in M7: *EGFR* (fold 19.79, *P* <1e-5), *ERBB4* (fold 19.16, *P* <1e-5), and *ERBB2*/*3* (fold 13.20, *P* <1e-5). Interestingly, this module also overlaps with genes that are upregulated in response to EGF signaling in HeLa cells (fold 6.87, *P* <1e-5) [63]. In our model system, we observed clear differential regulation of the EGF receptors. *ERBB2* and *ERBB3* are epigenetically and transcriptionally repressed, while *EGFR* is activated (see ‘Regulation of EMT signaling pathways is chromatin-mediated’). Repression of ErbB2/3 signaling coincides with the repression of many of its interaction partners [See Additional file 18: Figure S9]. Interestingly, among these repressed binding partners are other RTKs, including *FGFR2* and *FGFR3*. Further examination of M7 revealed an enrichment of signaling cascades that are downstream of cellular junctions, most significantly the focal adhesion pathway (fold 19.90, *P* 1.2e-68). Other over-represented cell adhesion pathways include integrins (fold 31.42, *P* <1e-5), adherens junctions (fold 27.61, *P* <1e-5), nectins (fold 87.22, *P* <1e-5), and tight junctions (fold 12.11, *P* <1e-5). Together, these results illustrate the co-regulation of EGF receptors, their downstream signaling pathways, and their transcriptional targets.

In summary, we find three modules (M1, M4, M7) within the EMT-network that correspond to signal transduction networks associated with TNF / TGFβ stimulation, as well as EGF signaling. Remarkably, we find that the same pathways associate with hubs of the EMT-network. Together, these results suggest that coordinated changes in chromatin maintain the activity of pathways associated with the response to TNF / TGFβ into the mesenchymal state. A plausible mechanism for how signaling from these pathways is integrated into a transcriptional response is provided by the remaining module, six.

### Transcriptional integration of upstream signaling

Examination of M6 revealed an enrichment for TFs and other nuclear proteins involved in cell-cycle regulation, chromatin modifications, and epigenetic regulation. GO-terms enriched in this module include ‘nucleus’ (fold 13.51, *P* <1e-16), ‘activating transcription factor binding’ (fold 30.19, *P* 4.7e-7), and ‘repressing transcription factor binding’ (fold 57.79, *P* 7.1e-12). Therefore, in contrast to the three upstream signaling modules, we refer to M6 as ‘downstream’. Interestingly, we observed enrichment of TNF-related regulators of NF-κB activity (fold 10.74, *P* 1.8e-30). This functionally links modules M6 and M4. A highly significant enrichment for TGFβ signaling (fold 34.64, *P* 2.5e-79), particularly through SMAD2 and 3 (fold 52.01, *P* 6.1e-51) indicates that M6 similarly associates with M1. Finally, the overrepresentation of EGF receptor signaling pathways from EGFR (fold 10.03, *P* <1e-5) and ERBB2/3 (fold 11.86, *P* 2.1e-05) suggests signaling from M7 to M6. There is also an over-representation of the MAPK targets and nuclear events mediated by MAP kinases in this module (fold 49.94, *P* <1e-5), as well as the inclusion of all genes in Reactome annotated as known regulators of the AP-1 family TFs (*P*<1e-5). In summary, we found evidence that M6 integrates signaling events from all three upstream modules.

We identified transcription factors within M6 that are also major hubs in the EMT-network (Figure 6) and hence are likely to mediate the transcriptional response. We found that *SMAD3*, *JUN*, *MYC*, and *RELA* satisfy these criteria. Interestingly, *JUN* and *MYC* are immediate early genes, while *SMAD3* and *RELA* are post-translationally activated in response to TGFβ and TNF, respectively. All four TFs reside in the EMT-GCs. Together, these data suggest sustained activation, coordination and maintenance of the early cytokine response pathways through concerted changes in histone modifications.

Furthermore, *JUN*, *MYC*, and *RELA* represent members of each of the transcription factor families identified in the enhancer analysis, which we implicate in our chromatin-mediated transcriptional feedback hypothesis (see ‘Links between enhancer clusters, gene clusters, and TFs, indicate a mechanism of chromatin-mediated transcriptional feedback’). Thus, we looked for evidence of regulatory loops within the EMT-network. To test this we examined the upstream modules for targets of AP-1, NF-κB, and c-Myc. Strikingly, we found enrichment of genes that are transcriptionally regulated by two AP-1 family members, *FOSL1* and *FOSL2* (fold 30.15, *P* <1e-5), in M1; enrichment of NF-κB target genes involved in the regulation of apoptosis (fold 219.36, *P* <1e-5) in M4; enrichment of targets of AP-1 (fold 2.60, *P* <1e-5) in M7; and enrichment of predicted NF-κB targets (fold 6.10, *P* <1e-5) in M6 itself. This implicates the AP-1 (which includes *JUN*) and NF-κB (which includes *RELA*) transcription factor families as positive transcriptional regulators of the upstream components of EMT-network.

There is also evidence that suggests an analogous, but inverted role for c-Myc (*MYC*). We found enrichment of genes that are downregulated by c-Myc in M1 (fold 10.39, *P* 2.0e-11), M6 (fold 14.66, *P* 2.84e-14), and M7 (fold 4.19, *P* 4.5e-9). This agrees with our previous results, which provide evidence for the repression of enhancers that bind c-Myc, the activation of genes in GC16 that are known to be repressed by c-Myc, and the repression of genes in GC15 that are activated by c-Myc. These data suggest opposing roles for AP-1 / NF-κB and c-Myc in the regulation of genes from the EMT-GCs. Overall, these results are consistent with the GO and pathway enrichment analyses of the EMT clusters, as well as the enhancer TFBS analysis.

## Conclusions

A rapidly growing body of research demonstrates that EMT is an epigenetically regulated process (for recent reviews see [64,65]). The known mechanisms of regulation involve miRNAs, chromatin structure, DNA methylation, and changes to histone modification levels. EMT in non-transformed cells has been likewise linked to remodeling of specific chromatin domains (that is, the so-called ‘LOCKs’) [9]. It was therefore plausible to hypothesize that genes involved in EMT are broadly coordinated through epigenetic mechanisms. We have made five key observations in support of this:

1. Genes known to be associated with the EMT phenotype are shown to have strong, specific, and highly similar differential chromatin profiles.

2. Epigenetic regulation at gene and enhancer loci linked to EMT is consistent in terms of chromatin activation, repression and differential gene expression.

3. Two distinct classes of enhancers associated with activated or repressed chromatin, are significantly enriched for binding sites of two different sets of TFs.

4. The upstream pathways and downstream targets of the TFs linked to activated enhancers (AP-1 and NF-κB family members) are enriched for genes with EMT-specific epigenetic profiles.

5. Network analysis of interactions among genes with EMT-specific epigenetic profiles highlights these TFs as protein-protein interaction hubs.

Therefore, epigenetic regulation of genes that drive EMT is coordinated and specific in our A549 model system. These findings link chromatin remodeling to shifts in cellular signaling networks. They are also consistent with a model of positive feedback that maintains the phenotypic switch (Figure 5A). The constitutive activation of NF-κB in our system and the extensive reprogramming at NF-κB target loci provide further support for this data-driven hypothesis.

Although we have been able to associate combinatorial epigenetic profiles with clear functional roles, our results do not address the specific cooperative mechanism of chromatin remodeling. However, we identified a number of candidate chromatin modifying enzymes that are differentially expressed. Upregulated chromatin modifiers include the histone deacetylase *HDAC9* (log2fc 3.53), methyltransferase *EZH2* (log2fc 1.13), and demethylases *JHDM1D* (log2fc 3.38) and *KDM1B* (log2fc 1.38). Downregulated enzymes include the deacetylase *HDAC1* (log2fc −1.15), methyltransferases *ELP3* (log2fc −0.92) and *NCOA2* (log2fc −1.43), and the demethylase *EHMT2* (log2fc −1.10). In addition, genes and enhancers with EMT-specific chromatin remodeling patterns are enriched for targets of specific chromatin remodeling complexes. For example, ENCODE-mapped Sin3a and HDAC2 binding sites are enriched in repressed enhancers. These factors have been implicated in EMT by a study that has shown that the master switch factors SNAI1 and SNAI2 recruit the Sin3a/HDAC1/HDAC2 complex to silence *CDH1* in EMT [66]. We also observe enrichments of known HDAC1 and HDAC2 targets among upregulated genes and within EMT-GCs. Consistently, we observe evidence for a decrease in HDAC1 and HDAC2 activity through the downregulation of *HDAC1* expression, and repression enhancers with HDAC2 binding sites. These associations point toward select chromatin modifying complexes and enzymes as likely epigenetic drivers of EMT.

We also found that chromatin modulates, and effectively maintains the activation of pathways involved in the response to TNF / TGFβ after prolonged stimulation with these cytokines. Surprisingly, many canonical immediate early response genes, such as *JUN*, remained active transcriptionally and epigenetically. Many of the pathways downstream of TNF / TGFβ show further evidence of chromatin-mediated transcriptional switching. Within the TGFβ signaling pathway we observe a striking bidirectional regulation of TGFβ superfamily cytokines, their receptors, and their downstream signaling components. We also see differential regulation of MAPK phosphatases and a pronounced switch in EGF receptors. Within these examples, genes that are upregulated often have the GC16 or GC19 activated epigenetic signature, while downregulated genes have the opposite GC15 repressed differential profile. These results are consistent with previous findings that EMT involves switches among receptor tyrosine kinases that activate the MAP-ERK pathway [67]. Thus, we conclude that modulation of critical pathways during EMT involves coordinated epigenetic activation and repression.

One of our most unexpected findings is that epigenetically active and repressed enhancer regions are enriched for the binding sites of two non-overlapping sets of specific TFs. This lends support to the model that chromatin and TF profiles jointly govern the locus specific regulation of gene expression. The magnitude of the differential epigenetic regulation that we observe at enhancers is in agreement with several studies that highlight the epigenetic plasticity of enhancers relative to promoters [24,31]. Our results suggest that global availability of TF binding sites at enhancers distinguish epithelial and mesenchymal phenotypes. Consistently, several studies have demonstrated the cell-type specificity of enhancers and TF binding patterns [68,69]. There is also evidence that the observed regulation of enhancers is specific to epithelial and mesenchymal phenotypes. For example, we linked FOXA1 and FOXA2 with enhancers that are repressed in EMT. These so-called ‘pioneer’ factors are believed to facilitate opening of chromatin at enhancers to enable lineage specific transcriptional regulation [70-72]. Interestingly, these TFs have been shown to promote the epithelial phenotype and block EMT in various systems [73-76].

In summary, we have shown extensive epigenetic reprogramming at both gene and enhancer loci between the end states of the EMT. Changes to chromatin states enable the constitutive activation of transcription factors (some of which are associated with an immediate early response), their upstream signaling pathways, and target enhancers. Based on these results we put forward a hypothesis in which EMT is driven in large part by chromatin-mediated activation of transcriptional positive feedback loops. The linchpins of this feedback are two TF families: AP-1 and NF-κB. Interestingly, of all gene clusters, GC15 and GC16 show the highest fractional composition of transcription factors, which includes a large number of AP-1 and NF-κB family members. This suggests that epigenetic reprogramming during EMT alters the transcriptional profile of the cell by broadly altering chromatin accessibility, and by regulating genes that directly mediate transcription–a potential feedback mechanism in itself. Together, our results suggest a high-level mechanism for how complex signaling networks can be coordinated during EMT, and cellular state transitions, generally.

## Methods

### Cell culture

NSCLC lines A549 were purchased from ATCC (Manassas, VA) and grown in DMEM (Mediatech, Manassas, VA), 10% FBS (Life Technologies, Grand Island, NY) and penicillin/streptomycin (Life Technologies). Spheroid (3D) cultures were resuspended in DMEM/10%FBS as 25000 cell aggregates using the hanging droplet technique. Newly formed spheroids were transferred onto polyhema plates containing DMEM/2% FBS to prevent aggregates from attaching to the dish. For EMT-induction, monolayer or spheroid cultures were incubated in DMEM/2% FBS and treated with vehicle or with TNF (10 ng/mL) and TGFβ (2 ng/mL) for 48 hours. The 2D and 3D cultures were then treated with vehicle or TNF and TGFβ a second time for an additional 48 hours. The samples were subsequently collected and subjected to RNA isolation or ChIP-seq. TGFβ (PHG 9204) and TNF (PHG 3015) were purchased from Life Technologies.

### ChIP-seq

Chromatin immunoprecipitation (IP) followed by sequencing (ChIP-seq) assays were performed in spheroid cultures only. TGFβ / TNF treated and control cells were cross-linked in 1% formaldehyde. The cross-linking reaction was quenched using 125 mM glycine, and the samples were collected for ChIP-seq analysis according to the Myers lab protocol as described in [77]. Approximately 1.2e7 cells were used per IP, and the DNA was sheared to approximately 400 bp fragments by sonication with a bioruptor. After DNA recovery, we used standard Illumina protocols and reagents to prepare the ChIP-seq library (Illumina 11257047 rev A). The antibodies used for IP are listed: H2A.Z (Abcam, ab4174), H3K4me1 (Active Motif, 39635), H3K4me2 (Active Motif, 39141), H3K4me3 (Active Motif, 39159), H3K27ac (Abcam, 4729), H3K27me2 (Active Motif, 39245), H3K27me3 (Active Motif, 39155), H3K14ac (Active Motif, 39599), H3K36me3 (Abcam, ab9050), H3K79me3 (Abcam, ab2621), H3K9ac (Active Motif, 39137), H3K9me1 (Active Motif, 39249), H3K9me3 (ab8898), HeR17me2asym (Abcam, ab8284), H4K8ac (Millipore, 17–10099), H4R3me2asym (Abcam, ab5823), H4K20me1 (Active Motif, 39175), pan-H3 (Active Motif, 39163).

### Microarray and gene expression analysis

Microarray analysis of gene expression was performed on technical duplicates of TGFβ / TNF treated and untreated cells in both two-dimensional and spheroid cultures. Total isolated mRNA was hybridized to Affymetrix U133 plus 2.0 microarrays. The raw data was analyzed using Bioconductor [78]. Background subtraction was performed using GCRMA. The Limma [79] package was used to perform differential expression analysis, in which a 5% FDR-adjusted *P* value cutoff was chosen.

Normalized expression values for all probes were propagated onto genes considered in this analysis. We used a comprehensive, but non-redundant, set of high-confidence protein-coding transcripts. We eliminated the majority of redundant transcripts coding for isoforms of a single gene, together with pseudo- and RNA-coding genes. For the full list of 20707 canonical transcripts represented by UCSC IDs [80] and gene symbols (HGNC) [see Additional file 8: Table S5]. Further, each gene was annotated with expression values from all probes that map to any of the genes’ transcripts and isoforms as defined by all the transcripts known to UCSC (July 2011). In analyses of differential gene expression the probe set with the largest log2 fold-change (log2fc) magnitude between treated and untreated samples has been chosen to represent a set of transcripts and was reported in Additional file 8: Table S5.

### Enhancer-associated histone modifications

Within our panel of epigenetic modifications we identified a subset of marks that are associated with enhancer activity. Marks that showed clear position-dependent correlation with either H3K4me1 or H3K27ac differential enrichment include: H3K4me2, H3K9ac, H3R17me2asym and H4K8ac [see Additional file 1: Figure S1]. Together with the initial two, these marks comprised our set of six enhancer-associated marks.

### ChIP-seq data processing

Images generated by the Illumina sequencer were initially processed using the Illumina pipeline. Sequences were mapped to the human reference genome, hg19 (GRCh37), using the BWA software [81] with all default options. In cases where a tag aligned to multiple sites the match with the smallest edit distance was chosen. In the event of an exact tie a single mapping site was randomly chosen. Sequences that fully or partially overlapped problematic regions were discarded. We defined problematic regions as those with known mapability issues, (for example, repetitive sequences (from the UCSC genome browser microsatellite track (downloaded July 8, 2011))) and genomic coordinates with high false positive rates of enrichments, as identified by [82]. All remaining mapped tags were extended to 200 bp in the 3’ direction to account of the expected length of nucleosome-bound DNA.

### Scaled differential enrichments

To generate chromatin enrichments the genome was segmented into 200 bp bins. The extended tags were assigned to each genomic bin they overlapped. The raw enrichment (RE) is simply the per-window overlap count. REs have been calculated for each of the mapped histone marks from both epithelial (3D untreated) and mesenchymal (3D treated) samples. To allow for comparisons of enrichment profiles between the epithelial (E) and mesenchymal (M) samples, we normalized pairs of REs for each histone modification or variant. We used an in-house implementation of the normalization procedure used in the DESeq algorithm [83] to calculate scale factors for each pair. Scaled enrichments (SE) were obtained by multiplying REs window-wise by the appropriate scale factors. Finally, we calculated scaled differential enrichments (SDE) by subtracting (for all histone modifications separately) the epithelial SE (ESE) from the mesenchymal MSE at each genomic window (that is, SDE = ESE − MSE).

### Definition of putative enhancer loci

We have adapted the methodology of [25] to locate putative enhancer sites using histone modifications. A set of initial putative loci was derived from the raw enrichments of two ‘core enhancer’ marks H3K27ac and H3K4me1 that have been previously shown to be sufficient to distinguish enhancers from other genomic elements. The SICER software [84] was used to call peaks of both marks in the epithelial and mesenchymal states, using corresponding panH3 samples as a control. Peak calls with gaps less than or equal to 600 bp were merged. The final calls were based on a FDR-corrected *P* value <0.01. These peaks were subsequently used to delineate enhancer regions. Potential enhancer sites were anchored on the window within a given peak call that had the maximum nominal enrichment of one of the two marks, corresponding to the mark for which the peak was called. Since enhancers discovered by profiling p300 occupancy have been shown to be depleted of H3K4me3, these anchor sites were filtered to exclude those that overlapped H3K4me3 SICER peaks (called in the same manner as H3K4me1 and H3K27ac). Finally, anchor sites based on H3K4me1 peaks that were within 1 kb of sites based on H3K27ac peaks were collapsed to the H3K27ac-based site. The 200bp sites were extended by 1000 bp at both ends resulting in set of 75,937 putative enhancers all 2200 bp in length.

### Filtering and gene assignment of enhancer loci

The initial set of 75,937 putative enhancers was further filtered to enrich for regions with significant epigenetic changes during EMT. We retained enhancers with a significant change for at least one ‘enhancer-associated’ histone modifications. The significance calls were based on a extreme-value null-model derived from the set of all enhancers. For each enhancer a single extreme-value is retained that corresponds to the largest magnitude of change in either the positive (gain) or negative (loss) direction. The details of how these changes are calculated at each enhancer are described in ‘Signal Quantification and Scaling’. The distribution of maximal magnitudes was represented through a kernel density estimate (Gaussian kernel, bandwidth 0.025). The left tail of this distribution was used to calculate a Gaussian null model of the noise regime of the differential signals. This Gaussian null model has parameters and, where $\hat{\mu }$is equal to the mode of the kernel density estimate, and $\hat{\sigma }$ is calculated using the following equation:

$$\hat{\sigma }=\sqrt{\frac{1}{n-1}\sum_{i}^{x_{i}\leq \hat{\mu }}{\left(x_{i}-\hat{\mu }\right)}^{2}}$$

Potential enhancers that had a *P* value >0.05 were filtered, yielding a final set of 30,681 putative differential enhancers. These enhancers were assigned to genes they likely regulate using a heuristic method described by [52]. Briefly, each gene was assigned a cis-region defined as the region from the given gene’s TSS to the neighboring TSSs in either direction, or 1 Mb if the nearest TSS is further than 1 Mb. Enhancers that fall within a gene’s cis-region are assigned to that gene.

### Differential epigenetic profiles

We calculated differential epigenetic profiles (DEP) at both gene and enhancer loci. We base the DEPs on scaled differential enrichments (SDEs, see ‘Scaled Differential Profiles’) for all mapped histone modifications at gene loci, and enhancer associated marks at putative enhancer loci. The calculation is a multistep procedure that results in a profile (fixed-sized feature vector) that summarizes the multivariate differences in histone modification levels between the paired samples at each locus. In the first step, gene loci are split into segments (see ‘Gene Segmentation’), while enhancers are kept whole. Next, within all segments, SDEs for each considered histone modification are quantified (see ‘Signal Quantification and Scaling’).

### Gene segmentation

The calculation of the raw epigenetic profile is based on four segments delineated for each gene. The sizes of all but one segment are fixed. The remaining one accommodates the variable length of genes. The fixed size segments are: promoter (PR), transcription start site (TSS) and gene start (GS). The whole gene (WG) segment is variable in size but is at least 1.2 kb long. We define the sizes and boundaries of segments based on windows, which have a fixed size of 200 bp and have boundaries that are independent of genomic landmarks such as TSSs. The location of the TSS defines the reference window, which together with its two adjacent windows, defines the TSS segment. The two remaining fixed-size segments, PR and GS, have a size of 25 windows (5 kb). The PR and GS segments are located immediately upstream and downstream, respectively, of the TSS segment, while the WG segment begins at the TSS reference window and extends 5 windows (1 kb) beyond the window containing the transcription termination site. Enhancers were treated as single-segment, contiguous 11-window (2,200 bp) regions (see ‘Enhancer Definition’).

### Signal quantification and scaling

The genome-wide scaled differential enrichments (SDEs) quantify epithelial to mesenchymal differences for each mark at 200 bp resolution across the genome. Each gene segment comprises a set of bookended windows [see Additional file 2: Figure S2]. For each histone modification, and within each segment, we reduce the SDE to two numeric values, which intuitively capture the level of gain and loss of the mark in the epithelial to mesenchymal direction. Strictly speaking, we independently calculate the absolute value of the sum of the positive (gain) and negative (loss) values of the SDE within a segment. Hence, we obtain a gain and loss value for all histone modifications within each segment of a gene (or an enhancer region). The differential epigenetic profile (DEP) of each gene (or enhancer) is a vector of gains and losses of multiple histone modifications at all segments (single segment for enhancers). In the case of gene loci we quantify all histone marks, and in the case of enhancer loci only the enhancer-associated modifications are quantified (see ‘Enhancer-Associated Histone Modifications’). DEPs are arranged into a DEP matrix individually for genes and enhancers (Figure 2A, [see Additional file 6: Figure S3 and Additional file 7: Table S4]). Each row represents a DEP for a gene (or enhancer) and each column represents a segment-mark-direction combination (features). Columns (features) were non-linearly scaled using the following equation:

$$z=\frac{2}{1+e^{\frac{-2x}{u}}}-1$$

Where, *z* is the scaled value, *x* is the raw value and *u* is the value of some upper percentile of all *v*alues of a feature. We have chosen the 95^th^ percentile. Intuitively, this corrects for differences in the dynamic range of changes to histone modification levels and for differences in segment size. Scaled values (DEP elements) are within the 0 to 1 range. The scaling is approximately linear for about 95% of the data points.

### Data integration

To enable a broad, systemic view of genes, pathways, and processes involved in EMT, we have integrated a number of publicly available datasets containing functional annotations and other types of information within a semantic framework. Our experimental data and computational results were also semantically encoded and made interoperable with the publicly available data. This connected resource has the form of a graph and can be flexibly queried across original datasets. External, publicly available, data have been retrieved as database dumps, files or batch queries to web services / servers depending on the design of the original resource. We have processed the raw files using Python scripts and transformed them into RDF-XML files. Within the RDF-XML files a subset of entities from the original resource are ‘encoded’ based on an in-house ontology. The full set of RDF-XML files has been loaded into the Sesame OpenRDF triple-store. We have chosen the Gremlin graph traversal language for most queries.

### Annotation with GO-terms

Each gene was comprehensively annotated with Gene Ontology terms combined from two primary annotation sources: EBI GOA (retrieved 20110905) and NCBI gene2go (retrieved September 4, 2011). These annotations were merged at the transcript cluster level (see ‘Microarray and Gene Expression Analysis’), which means that GO-terms associated with isoforms were propagated onto the canonical transcript. The translation from source IDs (UniProt IDs, and Entrez Gene IDs for EBI and NCBI respectively) onto UCSC IDs was based on the mappings provided by UCSC and Entrez and was done using an in-house probabilistic resolution method. Every protein-coding gene was re-annotated with terms from two GO-slims provided by the Gene Ontology consortium. The re-annotation procedure takes specific terms and translates them to generic ones. We used the map2slim tool and the two sets of generic terms: ‘PIR’ (Protein Informatics Resource) and ‘generic terms’. Besides GO, we have included two other major annotation sources: NCBI BioSystems, and the Molecular Signature Database 3.0 (MSigDB).

### Mining for genes associated with epithelial-mesenchymal transition

We attempted to construct a representative list of genes relevant to EMT. This list was obtained through a manual survey of relevant and recent literature. We extracted gene mentions from recent reviews on the epithelial-mesenchymal transition. A total of 142 genes were retrieved and successfully resolved to UCSC transcripts. The resulting list of protein-coding genes is available in Additional file 4: Table S2. A second set of genes associated with EMT was based on GO annotations. This set included all genes that were annotated with at least one term from a list of GO-terms clearly related to EMT [see Additional file 5: Table S3].

### Functional similarity scores

We developed a score to quantify functional similarity for any two sets of genes. Strictly speaking, the functional similarity score (FSS) measures the degree of overlap between the two lists of GO-terms enriched for the two sets. First, we obtain two lists of significantly enriched GO-terms for the two sets of genes. The enrichment *P* values were calculated using Fisher’s Exact Test and FDR-adjusted for multiple hypothesis testing. For each enriched term we also calculate the fold change (that is, whether it is enriched or depleted relative to the background frequency). The similarity between any two sets is given by:

$$\mathrm{FSS}\left(A,B\right)=\sum_{c}^{C}\log\left(p_{c}^{A}\times p_{c}^{B}\right)+\sum_{d}^{D}\log\left(p_{d}^{A}\times p_{d}^{B}\right)$$

where A and B are two lists of significantly enriched GO-terms (here FDR-corrected *P* <0.01). C and D are sets of GO-terms that are either enriched or depleted in both lists, but not enriched in A and depleted in B and vice-versa. Intuitively, this score increases for every significant term that is shared between two sets of genes, with the restriction that the term cannot be enriched in one, but depleted in the other cluster. If one of the sets of genes is a reference list of EMT-associated genes, this functional similarity score is, in general terms, a measure of relatedness to the functional aspects of EMT.

### Functional correlation matrix

The functional correlation matrix (FCM) (Figure 2B) contains functional similarity scores (FSS) for all pairs of gene clusters with the difference that enrichment (E) and depletion (D) scores are not summed but are shown separately. Each row represents a ‘source’ gene cluster while each column represents either the enrichment (E) or depletion (D) score with a ‘target’ cluster. The FSS is the sum of the enrichment and depletion scores, (that is, FSS = E + D). Columns are arranged numerically by cluster ID, rows are arranged by Ward hierarchical clustering using the cosine metric. The FCM and clustering dendrogram have been visualized in Java TreeView.

### Selection of optimal clustering

We have followed a heuristic benchmarking approach to select a suitable unsupervised clustering method to group genes based on differential epigenetic profiles, while maximizing the biological interpretability of DEPs. Because there is no correct solution to unsupervised machine learning tasks, we evaluated clustering solutions based on their interpretability in the domain of the epithelial-mesenchymal transition. Intuitively, a ‘good’ clustering method groups genes with similar functions together. Therefore, we expected a small number of the clusters to be enriched for genes related to the EMT process (see ‘Mining for Genes Associated with EMT’). However, such straightforward approach would have the drawback of being strongly biased towards what is known, whereas the goal of unsupervised machine learning is to uncover what is not. To alleviate this problem, rather than calculating enrichments for genes known to be involved in EMT, we calculate the FSS that measures the degree of functional similarity between a cluster and a reference set of genes associated with EMT. Our goal was to find a combination of gene segmentation, data scaling and machine learning algorithm that performs well in grouping functionally related genes together. We evaluated three markedly different unsupervised learning methods: hierarchical clustering, AutoSOME [33], and WGCNA [85]. We further profiled a number of ways to partition gene loci into segments, and three methods to scale the columns of the DEP matrix (no scaling, non-linear scaling, non-linear-scaling with detrending). Based on the distribution of EMT-similarity scores (preferred few highly enriched clusters) and a number of semi-quantitative indicators such as cluster size (preferred small enriched clusters), differential gene expression (preferred up or down regulated clusters) we chose a final combination of clustering algorithm: AutoSOME, segmentation approach (see ‘Gene and Enhancer Segmentation’), and scaling method (see ‘Signal Quantification and Scaling’).

### Clustering of gene and enhancer loci

DEP matrices (see ‘Signal Quantification and Scaling’) associated with each of the 20,707 canonical transcripts (genes) and each of the 30,681 final enhancers were clustered using AutoSOME with the following settings: -P -g10 -p0.05 -e200. The output of AutoSOME is a crisp assignment of genes (or enhancers) into clusters and each cluster contains genes (enhancers) with similar DEPs. For visualization, columns (features) were clustered using hierarchical Ward clustering and manually rearranged if necessary. The matrices were visualized in Java TreeView.

### Transcription factor binding sites within promoters and enhancers

Transcription factor binding sites were obtained from the ENCODE transcription factor ChIP track of the UCSC genome browser [86] (downloaded December 15, 2011). This dataset contains a total of 2,750,490 binding sites for 148 different factors pooled from variety of cell types from the ENCODE project. The enrichment of each transcription factor in each enhancer and gene cluster was calculated as the cardinality of the set of enhancers or promoters (5,400 bp, centered on the window containing the transcription start site) that have a nonzero overlap with a given set transcription factor binding sites. The significance of the enrichment was calculated using a one-tailed Fisher’s Exact Test (cluster membership versus TF enrichment).

### Protein-protein interaction networks

The source of protein-protein interactions (PPIs) within our integrated resource is STRING9 [87]. This database collates multiple smaller sources of PPIs, but also applies text-mining to discover interactions from literature and further gives confidence values to network edges. For the purpose of this work, we focused on experimentally determined physical interaction with a confidence cut-off of 400, which is also the default from the STRING9 website. We obtained identifier synonyms that enabled us to cross-reference the interactions with entities from the protein aliases file. We explored the interaction graph from each of our 20,707 reference genes, by traversing along the interactions that met the type and cut-off requirements. Genes that had at least one interaction were retained. This full interaction graph has been exported as a GraphML file for further analysis and visualization.

We have constructed two sub-networks that highlight the interactions within smaller sets of genes than the full STRING9-derived interactome. A subnetwork contains interactions only between genes that induce it. These inducing sets of genes have been obtained by expanding seed gene lists. We used two seeds: (1) gene lists that were based on EMT-related gene clusters and (2) a list of down-regulated genes. The expansion of seeds into inducing sets included all genes that interacted with at least two genes from the seed. In other words, all genes that mediated interactions between genes in the seed list were discovered and appended and formed the inducing set. Genes within the EMT-GCs (GC15, GC16, GC19) were merged together into a single seed gene list, which formed the basis of the EMT-network (Figure 6). The downregulated gene expression network [See Additional file 18: Figure S9] has been constructed analogously to the epigenetic one, with the alteration that the seed lists were obtained by taking genes below a log2 fold-change −2 cut off.

### Hubs and modules

Within each network (or sub-network) we identified hubs [88] and modules [89]. We have employed the PageRank algorithm to identify hubs. We have used the fast heuristic algorithm of Blondel *et al*. [90] to discover dense communities, or modules, within our protein-protein interaction networks. Intuitively, modules within a PPI graph are groups of highly interconnected genes. We used a version of the Blondel *et al.* algorithm that depends on a resolution parameter, which we fixed for all analyses to 1.66 to yield slightly simpler solutions (fewer modules) [91]. All PageRank scores and modules have been calculated within the Gephi software.

### Data access

Data have been submitted to GEO: SubSeries GSE42373, gene expression GSE42374, ChIP-seq GSE42375.

## Abbreviations

DEGs: Delayed early genes; DEP: Differential epigenetic profiles; DUSPs: Dual-specificity phosphatases; EMT: Epithelial-mesenchymal transition; EMT-GCs: Epithelial-mesenchymal-gene cluster; SS: Similarity scores; FCM: Functional correlation matrix; FSS: Functional similarity score; GC: Gene cluster; GS: Gene start; IEGs: Immediate early genes; IP: Immunoprecipitation; NSCLC: Non-small cell lung cancer; PPI: Protein-protein interaction; PR: Promoter; RE: Raw enrichment; SDE: Scaled differential enrichments; SE: Scaled enrichment; TF: Transcription factor; TSS: Transcription start site; WG: Whole genome.

## Competing interests

The authors declare that they have no competing interests.

## Authors’ contributions

MC and SAH performed the bioinformatic analyses and prepared the manuscript. NB and SC prepared spheroid cultures and performed ChIP-seq experiments. MK developed the experimental system and prepared spheroid cultures. DA prepared spheroid cultures and edited the manuscript. XX aided in the bioinformatic analyses. JW and LG prepared spheroid cultures. MWM, DRJ, and SB conceived and guided the study and helped prepare the manuscript. All authors read and approved the final manuscript.

## Supplementary Material

Additional file 1: Figure S1 Correlation of histone modifications at enhancers. (A) Correlation of histone modifications with H3K4me1 at putative enhancer loci. (B) Correlation of histone modifications with H3K27ac at putative enhancer loci. Click here for file

Additional file 2: Figure S2 Gene segmentation and differential signal quantification. Gene loci were segmented into four regions: promoter, transcription start site (TSS), gene start, and gene body. Within each segment, two values were computed for each mark: the sum of the differential gain in the mark and the sum of the differential loss in the mark (absolute value, mesenchymal minus epithelial). These values together form the differential epigenetic profile (DEP) for each gene. Enhancers were treated similarly; however, enhancer loci were not segmented. Click here for file

Additional file 3: Table S1 Literature-based list of genes associated with epithelial-mesenchymal transition (EMT). List of genes associated with EMT from a manual search of recent literature. Click here for file

Additional file 4: Table S2 List of GO-terms associated with epithelial-mesenchymal transition (EMT). List of GO-terms associated with EMT, mesenchymal-epithelial transition (MET), or the regulation thereof. Click here for file

Additional file 5: Table S3 Functional similarity scores of epithelial-mesenchymal transition-related gene clusters (EMT-GCs). For each of the EMT-GCs, this table presents the 9 most similar (in terms of the GO-based functional similarity score) clusters and reference lists of EMT-associated genes. Click here for file

Additional file 6: Figure S3 Epigenetic epithelial-mesenchymal transition-related gene cluster (EMT-GCs) (detailed). (A) Differential epigenetic profiles (DEP) of the EMT-related clusters. Same as Figure 3A, but the rows (epigenetic features) and columns (clusters) have been swapped to show all epigenetic features. The naming of each feature follows a convention. The first three segments separated by ‘_’ correspond to: gene segment: pr - promoter, ts - transcription start site, gs - gene start, gr - gene body. The following numbers correspond to clusters at different resolutions (fine, coarse). Click here for file

Additional file 7: Table S4 GO-terms most significantly enriched for GC16. A list of the most significantly enriched GO-terms in the epithelial-mesenchymal transition (EMT)-cluster 16, which has the highest functional similarity score to lists of EMT-associated genes. The enrichment *P* values were calculated using Fisher’s Exact Test and false discovery rate (FDR) corrected. Click here for file

Additional file 8: Table S5 Gene expression, cluster membership, and module membership. Click here for file

Additional file 9: Table S6 GO term enrichments for upregulated genes. Click here for file

Additional file 10: Figure S4 Clusters in the differential expression-epigenetic plane. Click here for file

Additional file 11: Table S7 EMT-GC MSigDB enrichments. Click here for file

Additional file 12: Table S8 Epithelial-mesenchymal transition-related gene cluster (EMT-GC) pathway enrichments. Click here for file

Additional file 13: Figure S5 Heat map of differential enhancer clusters. Heat map showing differential enhancer clusters that are either activated or repressed. These clusters generally show gain (G) or loss (L) across all marks, corresponding to activation or repression, respectively. While H3R17me2asym shows correlation with differential H3K27ac levels at enhancers, it has relatively little coherence across the globally activated and repressed clusters. Additionally, of the marks that correlate with differential H3K27ac or H3K4me1 levels at enhancers, H3R17me2asym shows the weakest correlation (Supplementary Figure S1). Click here for file

Additional file 14: Figure S6 Activation and repression of enhancers correlate with changes in gene expression. The plot shows the correlation between differential gene expression (log2 fold-change color) and the ‘activation’ Y-axis, and ‘repression’ X-axis of proximal enhancers. Each dot represents a gene. Its position in the X-Y plane indicates whether its proximal enhancers are rather ‘activated’ (dot close to Y) or ‘repressed’ (dot close to X). Click here for file

Additional file 15: Figure S7 AP-1 and c-Myc enrichment in gene clusters via enhancers. Association of (A) AP-1 and (B) c-Myc binding sites with gene clusters via enhancers. Enrichment of each factor’s binding sites (ENCODE) in the enhancers assigned to each gene cluster. Click here for file

Additional file 16: Figure S8 PCR of *MYC* from cells before and after induction of the epithelial-mesenchymal transition (EMT). Three dimensional cultures of A549 cells were left alone (No Add) or treated with TNF and TGFb (TNF/TGF) for ninety-six hours. Expression of c-Myc (*MYC*) was measured by QRT-PCR using *MYC*, forward 5’-TCAAGAGGCGAACACACAAC-3’ and reverse 5’-GGCCTTTTCATTGTTTTCCA-3 primers. *MYC* expression levels were normalized to *GAPDH* using forward 5’-GAAGGTGAAGGTCGGAGTC-3’ and reverse 5’-GAAGATGGTGATGGGATTTC-3’ primers. Results shown were calculated mean ± S.D, *p <0.05, of three independent experiments. Click here for file

Additional file 17: Table S9 Hubs in the epithelial-mesenchymal transition (EMT)-network. List of hubs in the protein-protein interaction network induced by genes from the EMT-GCs. For each hub gene, its epigenetic cluster, PageRank and module number is reported. Click here for file

Additional file 18: Figure S9 Protein-protein interaction (PPI) network induced by downregulated genes. This network contains genes that are four-fold or more downregulated and genes that mediate interactions between down-regulated genes. Color of nodes corresponds to differential gene expression (blue – down, red – up, white – no change). Size of nodes corresponds to the PageRank. Click here for file
