# Supplementary material for: Epigenetic coordination of signaling pathways during the epithelial-mesenchymal transition
Source: Epigenetics Chromatin. 2013 Sep 2;6:28. doi: 10.1186/1756-8935-6-28 (PMC3847279; doi:10.1186/1756-8935-6-28)
Supplement: Additional file 1: Figure S1 — Correlation of histone modifications at enhancers. (A) Correlation of histone modifications with H3K4me1 at putative enhancer loci. (B) Correlation of histone modifications with H3K27ac at putative enhancer loci. [file 1756-8935-6-28-S1.docx]

### Supplementary Figure S1: Correlation of histone modifications at enhancers


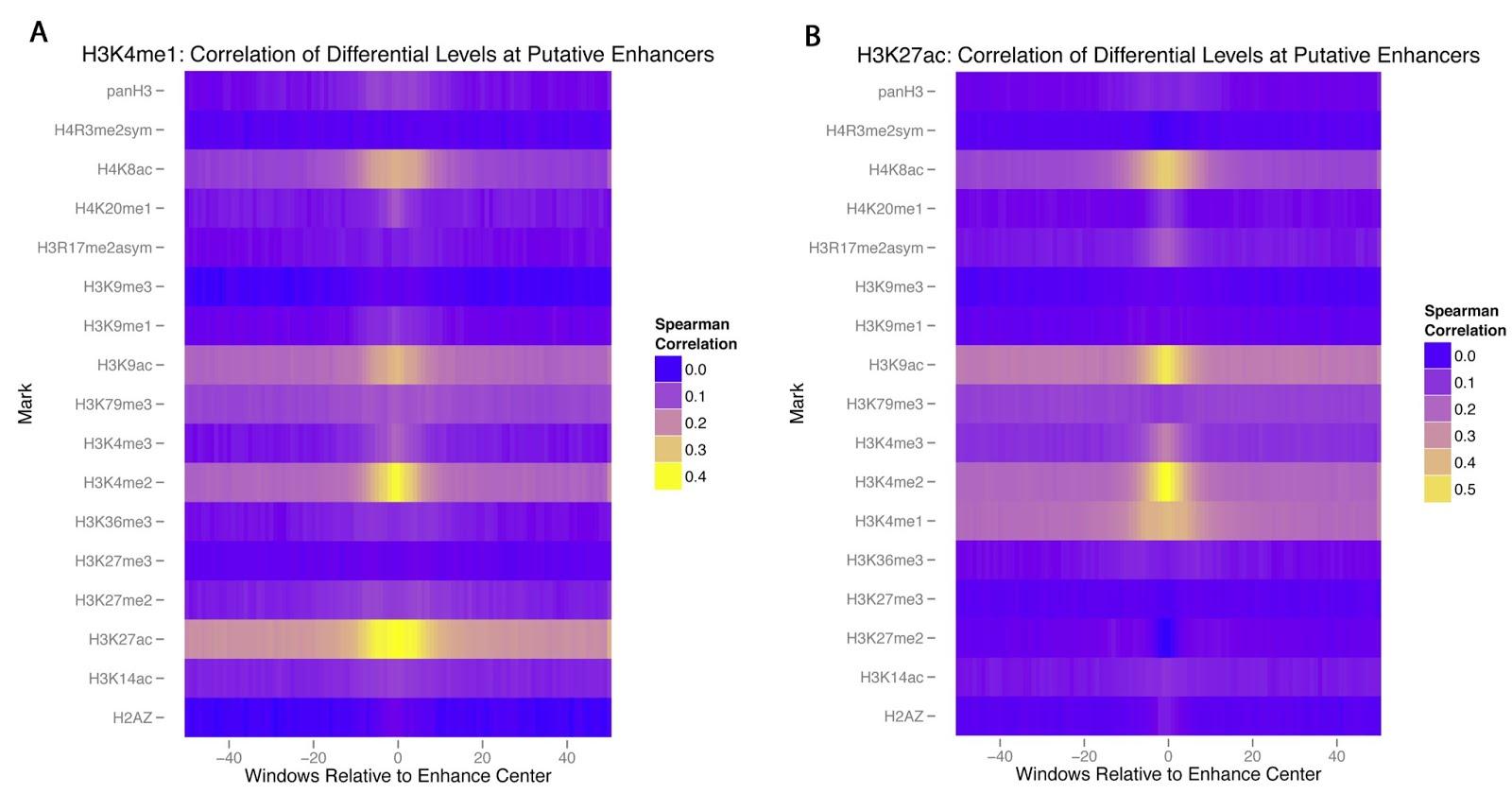


(A) Correlation of histone modifications with H3K4me1 at putative enhancer loci.

(B) Correlation of histone modifications with H3K27ac at putative enhancer loci.
