# Supplementary material for: Epigenetic coordination of signaling pathways during the epithelial-mesenchymal transition
Source: Epigenetics Chromatin. 2013 Sep 2;6:28. doi: 10.1186/1756-8935-6-28 (PMC3847279; doi:10.1186/1756-8935-6-28)
Supplement: Additional file 2: Figure S2 — Gene segmentation and differential signal quantification. Gene loci were segmented into four regions: promoter, transcription start site (TSS), gene start, and gene body. Within each segment, two values were computed for each mark: the sum of the differential gain in the mark and the sum of the differential loss in the mark (absolute value, mesenchymal minus epithelial). These values together form the differential epigenetic profile (DEP) for each gene. Enhancers were treated similarly; however, enhancer loci were not segmented. [file 1756-8935-6-28-S2.docx]

### Supplementary Figure S2: Gene segmentation and differential signal quantification


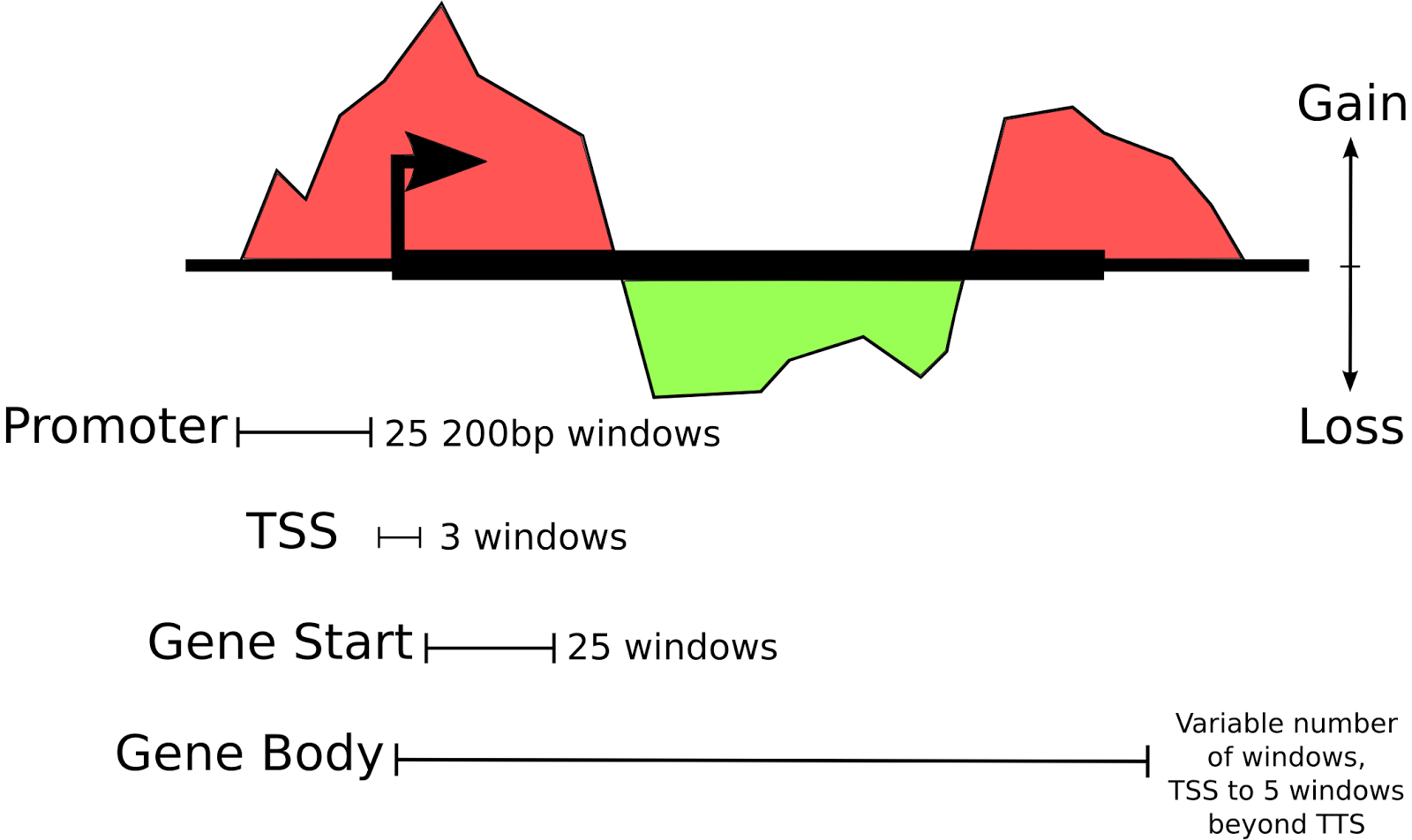


Gene segmentation and differential signal quantification. Gene loci were segmented into four regions: promoter, TSS, gene start, and gene body. Within each segment two values were computed for each mark: the sum of the differential gain in the mark, and the sum of the differential loss in the mark (absolute value, mesenchymal minus epithelial). These values together form the differential epigenetic profile (DEP) for each gene. Enhancers were treated similarly; however, enhancer loci were not segmented.
