# Supplementary material for: Epigenetic coordination of signaling pathways during the epithelial-mesenchymal transition
Source: Epigenetics Chromatin. 2013 Sep 2;6:28. doi: 10.1186/1756-8935-6-28 (PMC3847279; doi:10.1186/1756-8935-6-28)
Supplement: Additional file 3: Table S1 — Literature-based list of genes associated with epithelial-mesenchymal transition (EMT). List of genes associated with EMT from a manual search of recent literature. [file 1756-8935-6-28-S3.docx]

### Supplementary Table S1: literature-based list of genes associated with EMT

| uc002lqb.2_ELANE uc004dif.2_TIMP1 uc003fxn.3_DLG1 uc003stq.2_TSPAN13 uc003uhg.3_CD36 uc001ypn.2_AKT1 uc001ydu.2_GSC uc003vid.2_CAV2 uc003hzy.3_EGF uc001tbt.2_DCN uc001ddz.1_GADD45A uc002lwb.1_GADD45B uc002kwl.3_DSC2 uc003und.2_GNG11 uc003thr.2_INHBA uc003pel.2_PTP4A1 uc001wuq.2_SIP1 uc003gom.2_FGFBP1 uc002ewg.1_CDH1 uc001mhs.2_WEE1 uc002kwg.2_CDH2 uc001sjh.2_ERBB3 uc002mez.2_CRB3 uc003xqp.2_SNAI2 uc002flj.2_SNAI3 uc003fqq.2_HRG uc002xuz.2_SNAI1 uc003ban.1_PPPDE2 uc010gip.1_BMP7 uc003xbg.2_BMP1 uc002vfa.2_FN1 uc001opa.2_CCND1 uc003tzi.3_CLDN4 uc003cxy.3_MST1R uc003umy.1_TFPI2 uc001iou.2_VIM uc002mjq.1_ANGPTL4 uc003hju.1_SCARB2 uc002gfm.3_CLDN7 uc001iwt.3_ITGB1 | uc001htm.2_ACTA1  uc001phg.1_MMP10  uc001sga.2_ITGA5  uc002ilj.2_ITGB3  uc002uqj.1_COL3A1  uc001hhh.2_LAMB3  uc003edn.2_GSK3B  uc001nqi.2_TMEM132A  uc003uxt.2_SERPINE1  uc002qyj.2_SOX11  uc001ntl.2_AHNAK  uc001zkh.2_THBS1  uc001dab.2_INADL  uc003ung.1_COL1A2  uc010crj.2_NLK  uc003tzg.3_CLDN3  uc001atj.2_NPPB  uc001xjh.1_PLEK2  uc010dph.1_TCF4  uc003kbw.3_MAP1B  uc002ltt.3_TCF3  uc001hqd.3_PARP1  uc003mxp.1_DSP  uc002hus.2_IGFBP4  uc002vgj.3_IGFBP5  uc003hmd.2_FGF5  uc001ivs.3_ZEB1  uc003lmn.3_FGF1  uc002tvu.2_ZEB2  uc002tjb.2_IL1RN  uc003qon.3_ESR1  uc004baz.1_TMEFF1  uc002upq.2_ITGAV  uc002hxf.1_KRT14  uc004fkk.2_FLNA  uc003dhn.2_WNT5A  uc002fjq.2_FOXC2  uc003ukr.2_CLDN12  uc003lro.2_PDGFRB  uc001jrc.2_NODAL | uc001mef.2_ILK  uc002lcz.2_SMAD2  uc002aqj.2_SMAD3  uc003yvt.2_PTK2  uc001saa.1_KRT7  uc001xdw.2_DACT1  uc002hzl.1_STAT3  uc002uyw.1_FZD7  uc003lui.2_SPARC  uc004ezo.2_FHL1  uc009wtt.2_F11R  uc002upy.2_TFPI  uc003tqk.2_EGFR  uc002uqk.2_COL5A2  uc001jtj.2_NUDT13  uc004cfe.2_COL5A1  uc003jwu.2_OCLN  uc003ckr.2_CTNNB1  uc003qtm.3_PLG  uc003qcz.2_CTGF  uc003hra.2_SPP1  uc003ujx.2_STEAP1  uc001bfr.2_CDC42  uc002oqh.1_TGFB1  uc001hln.2_TGFB2  uc001xsc.2_TGFB3  uc010ree.1_WT1  uc003kgy.1_DHFR  uc003kii.3_VCAN  uc002iaz.2_EZH1  uc004dwf.2_MSN  uc003wfb.1_EZH2  uc003dnz.2_MITF  uc003xdh.1_LOXL2  uc003sum.2_TWIST1  uc001txt.2_PXN  uc001gsl.2_RGS2  uc003xiu.2_NRG1  uc002wnw.2_JAG1  uc004akr.2_VPS13A | uc011kgj.1_YWHAG  uc002rsq.2_MTA3  uc001ecb.2_WNT2B  uc003axf.2_PDGFB  uc003spw.2_RAC1  uc003wsi.2_CLDN23  uc003dwt.1_CD47  uc001qjk.2_WNT5B  uc001pgx.2_BIRC3  uc003vrz.2_CALD1  uc002afz.2_CCNB2  uc003iwi.2_CASP3  uc003umu.1_CALCR  uc004chz.2_NOTCH1  uc003yzo.1_SCRIB  uc001epq.2_BCL9  uc002xqz.2_MMP9  uc001bdh.2_CAMK2N1  uc002ehz.3_MMP2  uc001phj.1_MMP3  uc001oxe.2_WNT11  uc002lit.1_BCL2 |
| --- | --- | --- | --- |

List of genes associated with EMT from a manual search of recent literature.
