# Supplementary material for: Epigenetic coordination of signaling pathways during the epithelial-mesenchymal transition
Source: Epigenetics Chromatin. 2013 Sep 2;6:28. doi: 10.1186/1756-8935-6-28 (PMC3847279; doi:10.1186/1756-8935-6-28)
Supplement: Additional file 4: Table S2 — List of GO-terms associated with epithelial-mesenchymal transition (EMT). List of GO-terms associated with EMT, mesenchymal-epithelial transition (MET), or the regulation thereof. [file 1756-8935-6-28-S4.docx]

### Supplementary Table S2: list of GO-terms associated with EMT

| **Accession** | **GO-term** |
| --- | --- |
| go:0060738  go:0003198  go:0001837  go:0060639  go:0060940  go:0072036  go:0044334  go:0060231  go:0060638  go:0060886  go:0072285  go:0003337  go:0061111  go:2000084  go:0010719  go:0003339  go:0010718  go:0060317  go:0060617  go:0060637  go:0060496  go:0060521  go:0003340  go:0060522  go:0060640  go:0060665  go:0072108  go:0060684  go:0061271  go:2000085  go:0003201  go:0060683  go:0010717  go:0061261  go:0060739  go:2000086 | epithelial-mesenchymal signaling involved in prostate gland development epithelial to mesenchymal transition involved in endocardial cushion formation epithelial to mesenchymal transition positive regulation of salivary gland formation by mesenchymal-epithelial signaling epithelial to mesenchymal transition involved in cardiac fibroblast development mesenchymal to epithelial transition involved in renal vesicle formation canon Wnt receptor signaling pathway involved in positive regulation of epithelial to mesenchymal transition mesenchymal to epithelial transition mesenchymal-epithelial cell signaling clearance of cells from fusion plate by epithelial to mesenchymal transition mesenchymal to epithelial transition involved in metanephric renal vesicle formation mesenchymal to epithelial transition involved in metanephros morphogenesis epithelial-mesenchymal cell signaling involved in lung development regulation of mesenchymal to epithelial transition involved in mesonephros morphogenesis negative regulation of epithelial to mesenchymal transition regulation of mesenchymal to epithelial transition involved in metanephros morphogenesis positive regulation of epithelial to mesenchymal transition cardiac epithelial to mesenchymal transition positive regulation of mammary placode formation by mesenchymal-epithelial signaling positive regulation of lactation by mesenchymal-epithelial cell signaling mesenchymal-epithelial cell signaling involved in lung development mesenchymal-epithelial cell signaling involved in prostate induction negative regulation of mesenchymal to epithelial transition involved in metanephros morphogenesis inductive mesenchymal to epithelial cell signaling positive regulation of dentine-containing tooth bud formation by mesenchymal-epithelial signaling regulation of branching involved in salivary gland morphogenesis by mesenchymal-epithelial signaling positive regulation of mesenchymal to epithelial transition involved in metanephros morphogenesis epithelial-mesenchymal cell signaling mesenchymal to epithelial transition involved in mesonephric renal vesicle formation negative regulation of mesenchymal to epithelial transition involved in mesonephros morphogenesis epithelial to mesenchymal transition involved in coronary vasculature morphogenesis regulation of branching involved in salivary gland morphogenesis by epithelial-mesenchymal signaling regulation of epithelial to mesenchymal transition mesenchymal to epithelial transition involved in mesonephros morphogenesis mesenchymal-epithelial cell signaling involved in prostate gland development positive regulation of mesenchymal to epithelial transition involved in mesonephros morphogenesis |

List of GO-terms associated with EMT, MET, or the regulation thereof.
