# Supplementary material for: Epigenetic coordination of signaling pathways during the epithelial-mesenchymal transition
Source: Epigenetics Chromatin. 2013 Sep 2;6:28. doi: 10.1186/1756-8935-6-28 (PMC3847279; doi:10.1186/1756-8935-6-28)
Supplement: Additional file 5: Table S3 — Functional similarity scores of epithelial-mesenchymal transition-related gene clusters (EMT-GCs). For each of the EMT-GCs, this table presents the 9 most similar (in terms of the GO-based functional similarity score) clusters and reference lists of EMT-associated genes. [file 1756-8935-6-28-S5.docx]

### Supplementary Table S3: functional similarity scores of EMT-GCs

| **cluster** | **cluster or list of EMT-associated genes** | **functional similarity score** |
| --- | --- | --- |
| GC15 | emtgenes_go emtgenes_lit GC16 GC04 GC19 GC01 GC18 GC03 GC09 | 205.761381346 106.772377206 76.4678281474 16.8759217647 16.1532919508 15.1664813866 14.0480186433 6.2439210232 6.03622128247 |
| GC16 | emtgenes_go emtgenes_lit GC19 GC09 GC01 GC04 GC18 GC15 GC02 | 1231.53358821 918.063894538 287.117110889 109.074387433 107.892455724 84.9963920061 80.9303836225 76.4678281474 74.707400527 |
| GC19 | emtgenes_go emtgenes_lit GC16 GC09 GC01 GC14 GC18 GC10 GC04 | 636.878772995 435.606099481 287.117110889 120.795771998 101.098122035 81.3221269711 69.6946488352 53.4902402939 36.8951867868 |

For each of the EMT-GCs, this table presents the 9 most similar (in terms of the GO-based functional similarity score) clusters and reference lists of EMT-associated genes.
