# Supplementary material for: Epigenetic coordination of signaling pathways during the epithelial-mesenchymal transition
Source: Epigenetics Chromatin. 2013 Sep 2;6:28. doi: 10.1186/1756-8935-6-28 (PMC3847279; doi:10.1186/1756-8935-6-28)
Supplement: Additional file 6: Figure S3 — Epigenetic epithelial-mesenchymal transition-related gene cluster (EMT-GCs) (detailed). (A) Differential epigenetic profiles (DEP) of the EMT-related clusters. Same as Figure 3A, but the rows (epigenetic features) and columns (clusters) have been swapped to show all epigenetic features. The naming of each feature follows a convention. The first three segments separated by ‘_’ correspond to: gene segment: pr - promoter, ts - transcription start site, gs - gene start, gr - gene body. The following numbers correspond to clusters at different resolutions (fine, coarse). [file 1756-8935-6-28-S6.docx]

### Supplementary Figure S3: Epigenetic EMT-related gene cluster (detailed)

**A
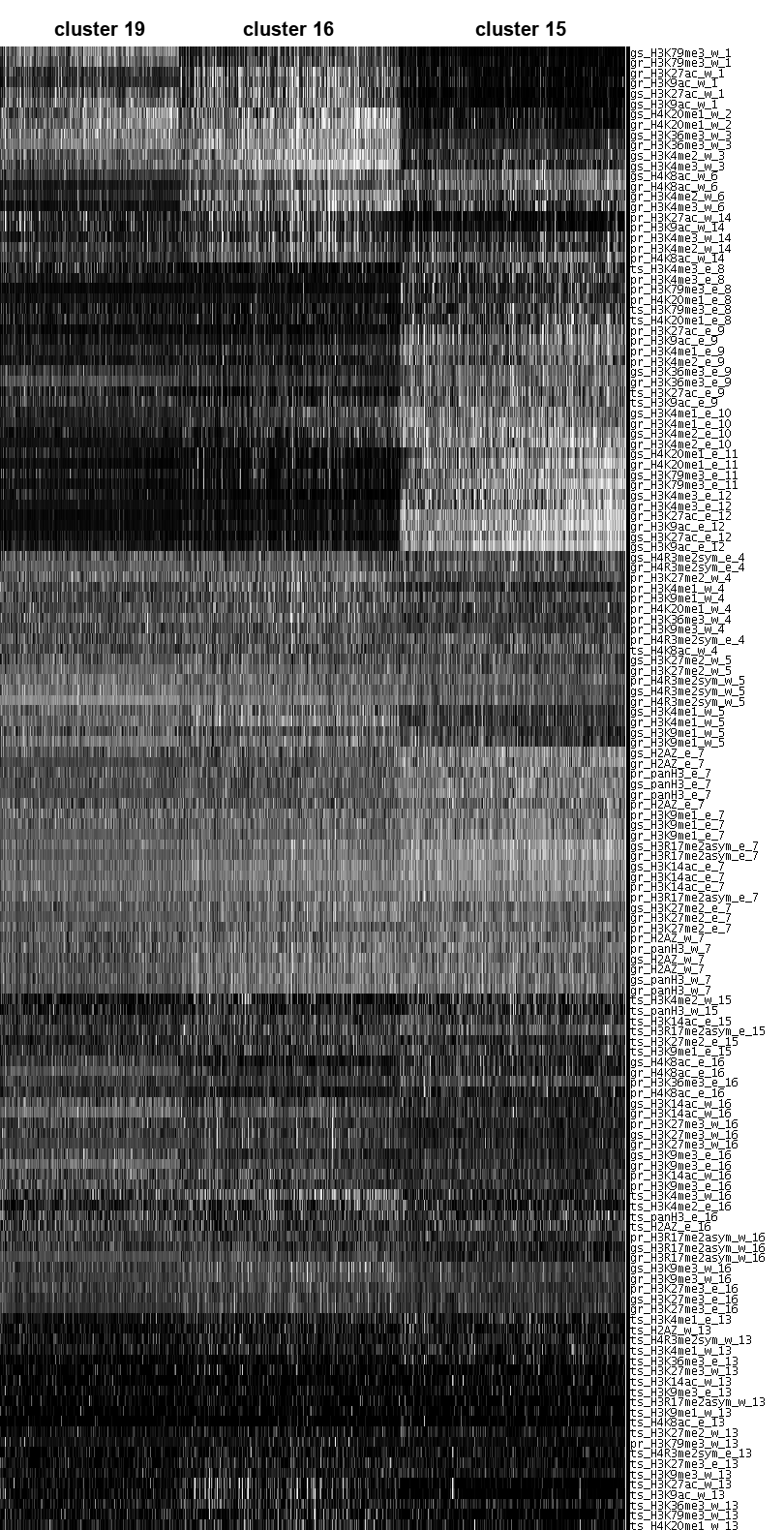
**

**B**
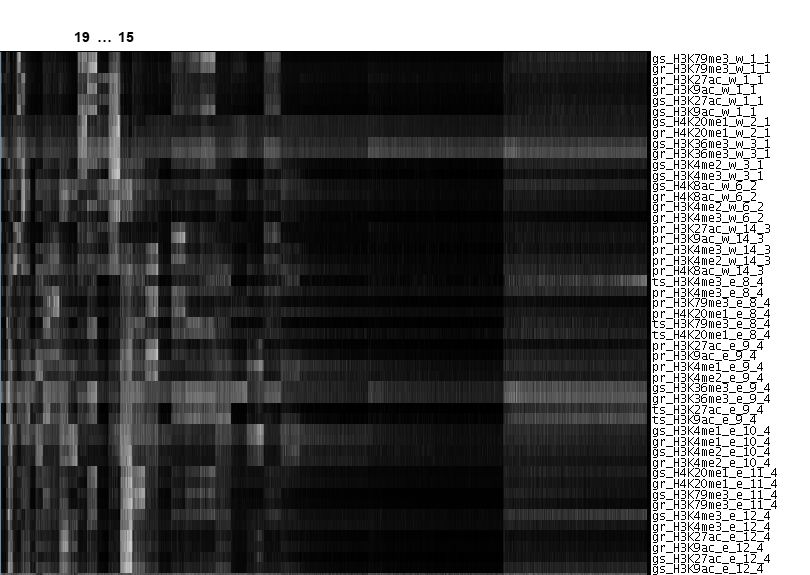


(A) Differential epigenetic profiles (DEP) of the EMT-related clusters. Same as Figure 3A, but the rows (epigenetic features) and columns (clusters) have been swapped to show all epigenetic features. The naming of each feature follows a convention. The first 3 segments separated by “_” correspond to: gene segment: pr - promoter, ts - transcription start site, gs - gene start, gr - gene body. The following numbers correspond to clusters at different resolutions (fine, coarse).
