# Supplementary material for: Epigenetic coordination of signaling pathways during the epithelial-mesenchymal transition
Source: Epigenetics Chromatin. 2013 Sep 2;6:28. doi: 10.1186/1756-8935-6-28 (PMC3847279; doi:10.1186/1756-8935-6-28)
Supplement: Additional file 7: Table S4 — GO-terms most significantly enriched for GC16. A list of the most significantly enriched GO-terms in the epithelial-mesenchymal transition (EMT)-cluster 16, which has the highest functional similarity score to lists of EMT-associated genes. The enrichment P values were calculated using Fisher’s Exact Test and false discovery rate (FDR) corrected. [file 1756-8935-6-28-S7.docx]

### Supplementary Table S4: GO-terms most significantly enriched for GC16

| **go-term** | **description** | **FDR p-value** |
| --- | --- | --- |
| go:0032502 go:0008219 go:0006950 go:0008283 go:0050896 go:0009987 go:0007155 go:0030154 go:0005515 go:0048856 go:0007165 go:0048646 go:0006928 go:0007154 go:0048870 go:0005615 go:0002376 go:0040011 go:0043066 go:0007568 go:0001525 go:0001816 go:0008285 go:0005576 go:0008150 go:0042060 | developmental process cell death response to stress cell proliferation response to stimulus cellular process cell adhesion cell differentiation protein binding anatomical structure dev. signal transduction anat. structure form. involved in morph. cellular component movement cell communication cell motility extracellular space immune system process locomotion negative regulation of apopt. aging angiogenesis cytokine production negative regulation of cell prolif. extracellular region biological_process wound healing | 0 0 0 0.000000000000001 0.000000000000002 0.000000000000003 0.000000000000019 0.000000000000047 0.000000000000329 0.000000000003768 0.000000000004093 0.00000000000842 0.000000000071532 0.000000000202326 0.000000000209765 0.000000000643198 0.000000003815827 0.00000003046501 0.000000230123362 0.000000828426091 0.000001219237428 0.000003457013022 0.000005147505251 0.000005614032241 0.000007644147652 0.000010570630365 |

A list of the most significantly enriched GO-terms in the EMT-cluster 16, which has the highest functional similarity score to lists of EMT-associated genes. The enrichment p-values were calculated using Fisher’s Exact Test and FDR corrected.
