# Supplementary figures and images for: Epigenetic coordination of signaling pathways during the epithelial-mesenchymal transition
Source: Epigenetics Chromatin. 2013 Sep 2;6:28. doi: 10.1186/1756-8935-6-28 (PMC3847279; doi:10.1186/1756-8935-6-28)

###
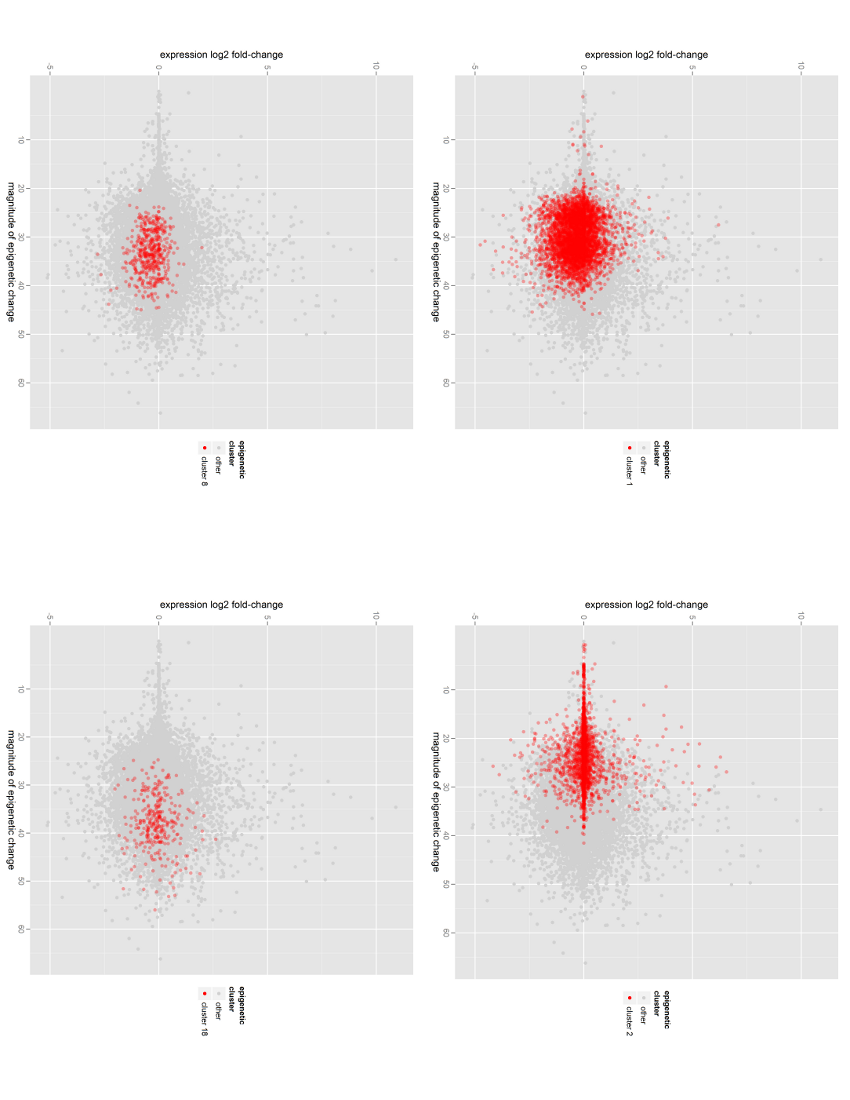
Supplementary Figure S4: Clusters in the differential expression-epigenetic plan

Supplement: Additional file 10: Figure S4 — Clusters in the differential expression-epigenetic plane. [file 1756-8935-6-28-S10.docx]
