# Supplementary material for: Epigenetic coordination of signaling pathways during the epithelial-mesenchymal transition
Source: Epigenetics Chromatin. 2013 Sep 2;6:28. doi: 10.1186/1756-8935-6-28 (PMC3847279; doi:10.1186/1756-8935-6-28)
Supplement: Additional file 13: Figure S5 — Heat map of differential enhancer clusters. Heat map showing differential enhancer clusters that are either activated or repressed. These clusters generally show gain (G) or loss (L) across all marks, corresponding to activation or repression, respectively. While H3R17me2asym shows correlation with differential H3K27ac levels at enhancers, it has relatively little coherence across the globally activated and repressed clusters. Additionally, of the marks that correlate with differential H3K27ac or H3K4me1 levels at enhancers, H3R17me2asym shows the weakest correlation (Supplementary Figure S1). [file 1756-8935-6-28-S13.docx]

### Supplementary Figure S5: Heat map of differential enhancer clusters


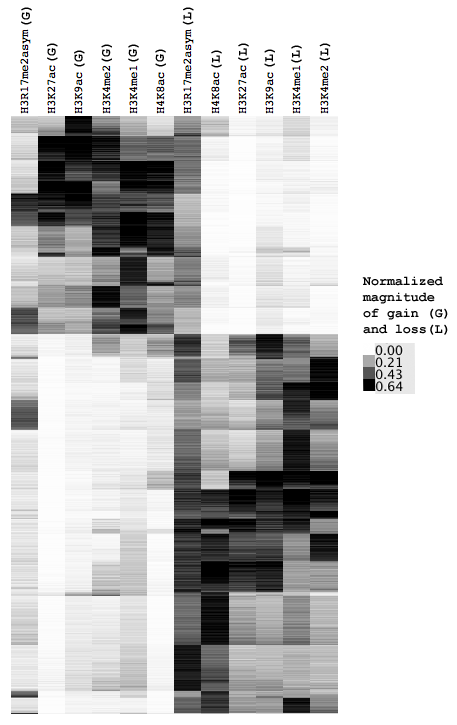


Heat map showing differential enhancer clusters that are either activated or repressed. These clusters generally show gain (G) or loss (L) across all marks, corresponding to activation or repression, respectively. While H3R17me2asym shows correlation with differential H3K27ac levels at enhancers, it has relatively little coherence across the globally activated and repressed clusters. Additionally, of the marks that correlate with differential H3K27ac or H3K4me1 levels at enhancers, H3R17me2asym shows the weakest correlation (Supplementary Figure S1).
