# Supplementary material for: Epigenetic coordination of signaling pathways during the epithelial-mesenchymal transition
Source: Epigenetics Chromatin. 2013 Sep 2;6:28. doi: 10.1186/1756-8935-6-28 (PMC3847279; doi:10.1186/1756-8935-6-28)
Supplement: Additional file 14: Figure S6 — Activation and repression of enhancers correlate with changes in gene expression. The plot shows the correlation between differential gene expression (log2 fold-change color) and the ‘activation’ Y-axis, and ‘repression’ X-axis of proximal enhancers. Each dot represents a gene. Its position in the X-Y plane indicates whether its proximal enhancers are rather ‘activated’ (dot close to Y) or ‘repressed’ (dot close to X). [file 1756-8935-6-28-S14.docx]

### Supplementary Figure S6: Activation and repression of enhancers correlates with changes in gene expression


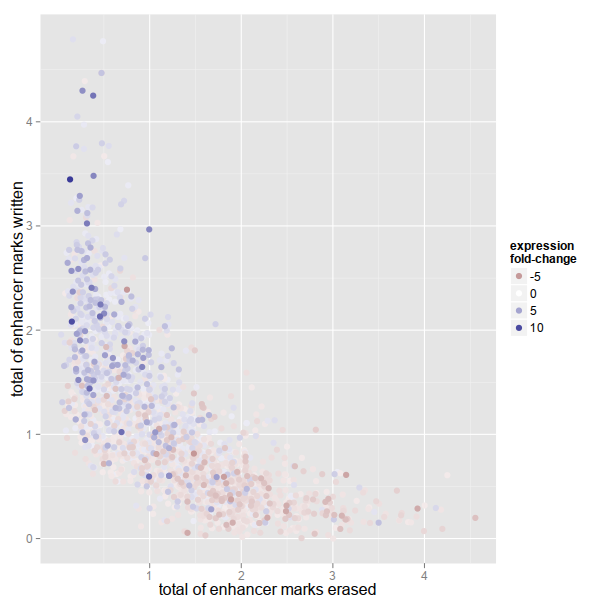


The plot shows the correlation between differential gene expression (log2 fold-change color) and the “activation” Y-axis, and “repression” X-axis of proximal enhancers. Each dot represents a gene. Its position in the X-Y plane indicates whether its proximal enhancers are rather “activated” dot close to Y or “repressed” dot close to X.
