# Supplementary material for: Epigenetic coordination of signaling pathways during the epithelial-mesenchymal transition
Source: Epigenetics Chromatin. 2013 Sep 2;6:28. doi: 10.1186/1756-8935-6-28 (PMC3847279; doi:10.1186/1756-8935-6-28)
Supplement: Additional file 15: Figure S7 — AP-1 and c-Myc enrichment in gene clusters via enhancers. Association of (A) AP-1 and (B) c-Myc binding sites with gene clusters via enhancers. Enrichment of each factor’s binding sites (ENCODE) in the enhancers assigned to each gene cluster. [file 1756-8935-6-28-S15.docx]

### Supplementary Figure S7: AP-1 and c-Myc binding site enrichment in gene clusters via enhancers


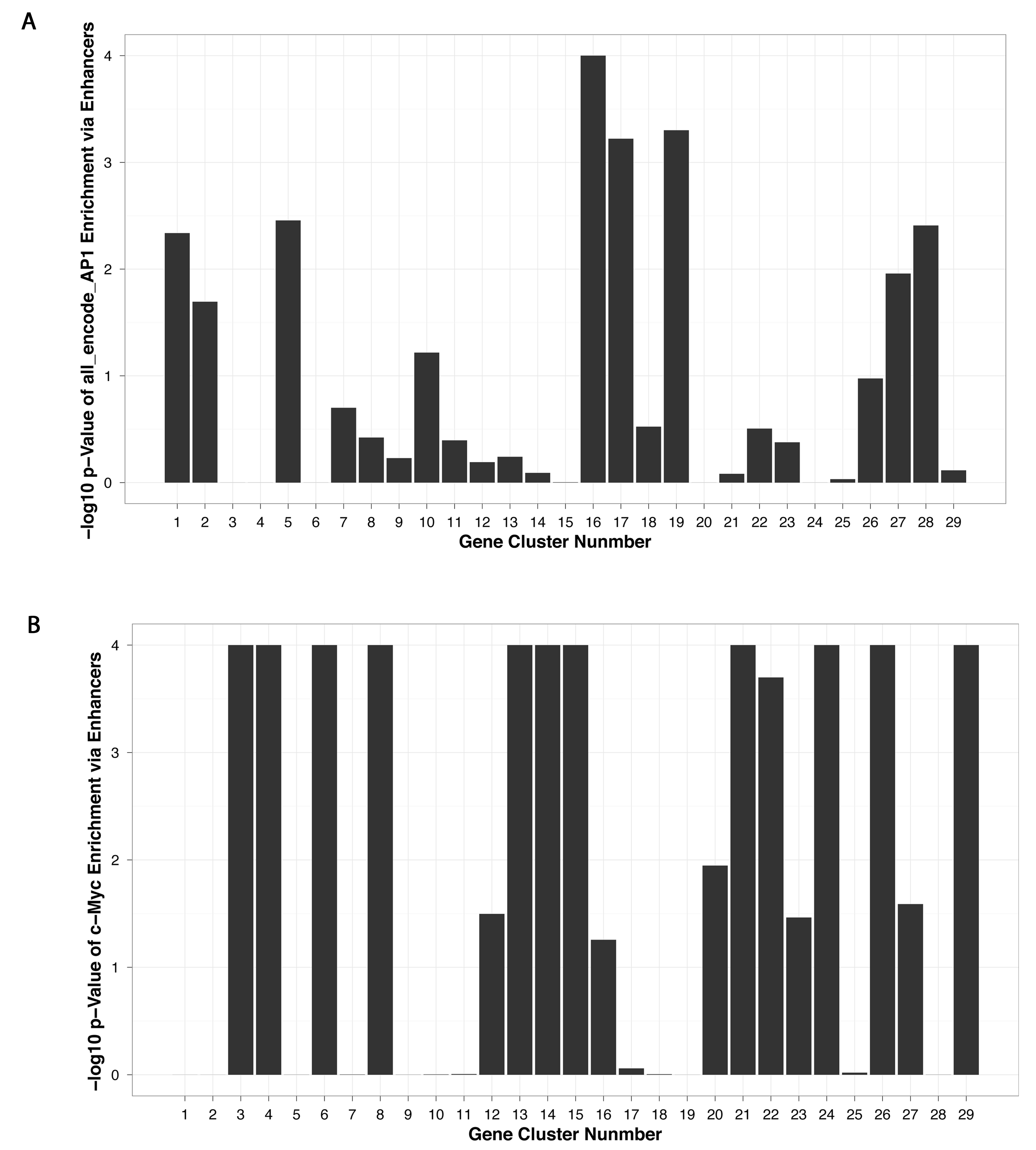


Association of (A) AP-1 and (B) c-Myc binding sites with gene clusters via enhancers. Enrichment of each factor’s binding sites (ENCODE) in the enhancers assigned to each gene cluster.
