# Supplementary material for: Epigenetic coordination of signaling pathways during the epithelial-mesenchymal transition
Source: Epigenetics Chromatin. 2013 Sep 2;6:28. doi: 10.1186/1756-8935-6-28 (PMC3847279; doi:10.1186/1756-8935-6-28)
Supplement: Additional file 16: Figure S8 — PCR of MYC from cells before and after induction of the epithelial-mesenchymal transition (EMT). Three dimensional cultures of A549 cells were left alone (No Add) or treated with TNF and TGFb (TNF/TGF) for ninety-six hours. Expression of c-Myc (MYC) was measured by QRT-PCR using MYC, forward 5’-TCAAGAGGCGAACACACAAC-3’ and reverse 5’-GGCCTTTTCATTGTTTTCCA-3 primers. MYC expression levels were normalized to GAPDH using forward 5’-GAAGGTGAAGGTCGGAGTC-3’ and reverse 5’-GAAGATGGTGATGGGATTTC-3’ primers. Results shown were calculated mean ± S.D, *p <0.05, of three independent experiments. [file 1756-8935-6-28-S16.docx]

### Supplementary Figure S8: PCR of *MYC* in before and after induction of EMT


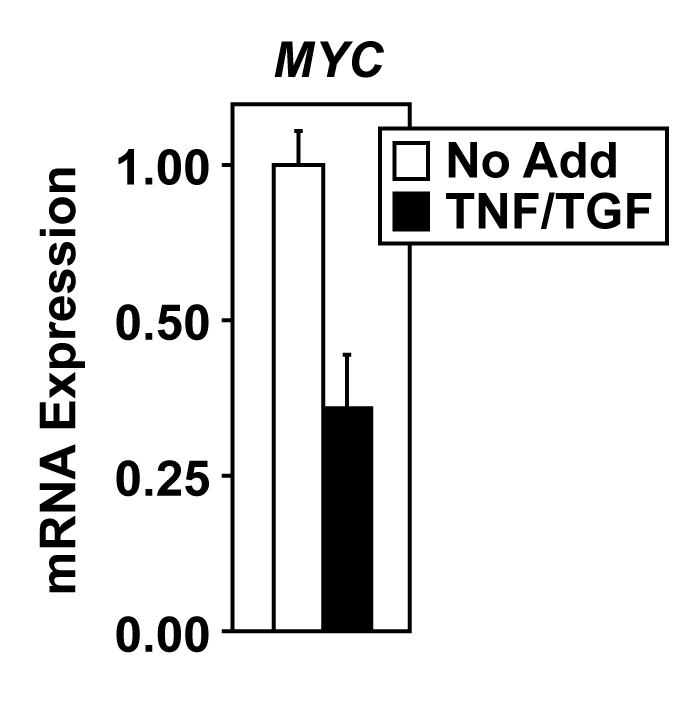


Three dimensional cultures of A549 cells were left alone (No Add) or treated with TNF and TGFb (TNF/TGF) for ninety-six hours. Expression of c-Myc (*MYC*) was measured by QRT-PCR using *MYC*, forward 5′-TCAAGAGGCGAACACACAAC-3′ and reverse 5′-GGCCTTTTCATTGTTTTCCA-3 primers. *MYC* expression levels were normalized to *GAPDH* using forward 5’-GAAGGTGAAGGTCGGAGTC-3’ and reverse 5’-GAAGATGGTGATGGGATTTC-3’ primers. Results shown were calculated mean ± S.D, *p <0.05, of three independent experiments.
