# Supplementary material for: Epigenetic coordination of signaling pathways during the epithelial-mesenchymal transition
Source: Epigenetics Chromatin. 2013 Sep 2;6:28. doi: 10.1186/1756-8935-6-28 (PMC3847279; doi:10.1186/1756-8935-6-28)
Supplement: Additional file 17: Table S9 — Hubs in the epithelial-mesenchymal transition (EMT)-network. List of hubs in the protein-protein interaction network induced by genes from the EMT-GCs. For each hub gene, its epigenetic cluster, PageRank and module number is reported. [file 1756-8935-6-28-S17.docx]

### Supplementary Table S9: Hubs in the EMT-network

| **gene** | **cluster** | **PageRank** | **module** |
| --- | --- | --- | --- |
| *ACTB CTNNB1 PRKCA EGFR SMAD3 JUN RELA RAC1 ABL1 IKBKG CDC42 NFKB1 PTK2 MYC RHOA HSPA1A UBC RXRA YWHAQ CAV1* | GC15 GC19 GC19 GC16 GC16 GC16 GC16 GC16 GC19 GC15 GC19 GC19 GC19 GC15 GC19 GC15 GC31 GC15 GC19 GC16 | 0.009812514608410358 0.008137655641433131 0.007567603554185837 0.00740360591481466 0.007284068183775619 0.006856907936399415 0.006692624429961651 0.006048362600922968 0.005567066216904118 0.005476110163224449 0.005377974924832943 0.0047451253191954 0.004702671000409733 0.004601641500311377 0.004601392423991404 0.004059409863611812 0.004015419479797583 0.003979052322132958 0.0037654930457035733 0.0037148072527496195 | 7 6 7 7 6 6 6 7 7 4 7 6 7 6 7 0 6 6 6 7 |

List of hubs in the protein-protein interaction network induced by genes from the EMT-GCs. For each hub gene its epigenetic cluster, PageRank and module number is reported.
