# Supplementary material for: Epigenetic coordination of signaling pathways during the epithelial-mesenchymal transition
Source: Epigenetics Chromatin. 2013 Sep 2;6:28. doi: 10.1186/1756-8935-6-28 (PMC3847279; doi:10.1186/1756-8935-6-28)
Supplement: Additional file 18: Figure S9 — Protein-protein interaction (PPI) network induced by downregulated genes. This network contains genes that are four-fold or more downregulated and genes that mediate interactions between down-regulated genes. Color of nodes corresponds to differential gene expression (blue – down, red – up, white – no change). Size of nodes corresponds to the PageRank. [file 1756-8935-6-28-S18.docx]

### Supplementary Figure S9: PPI network induced by down-regulated genes


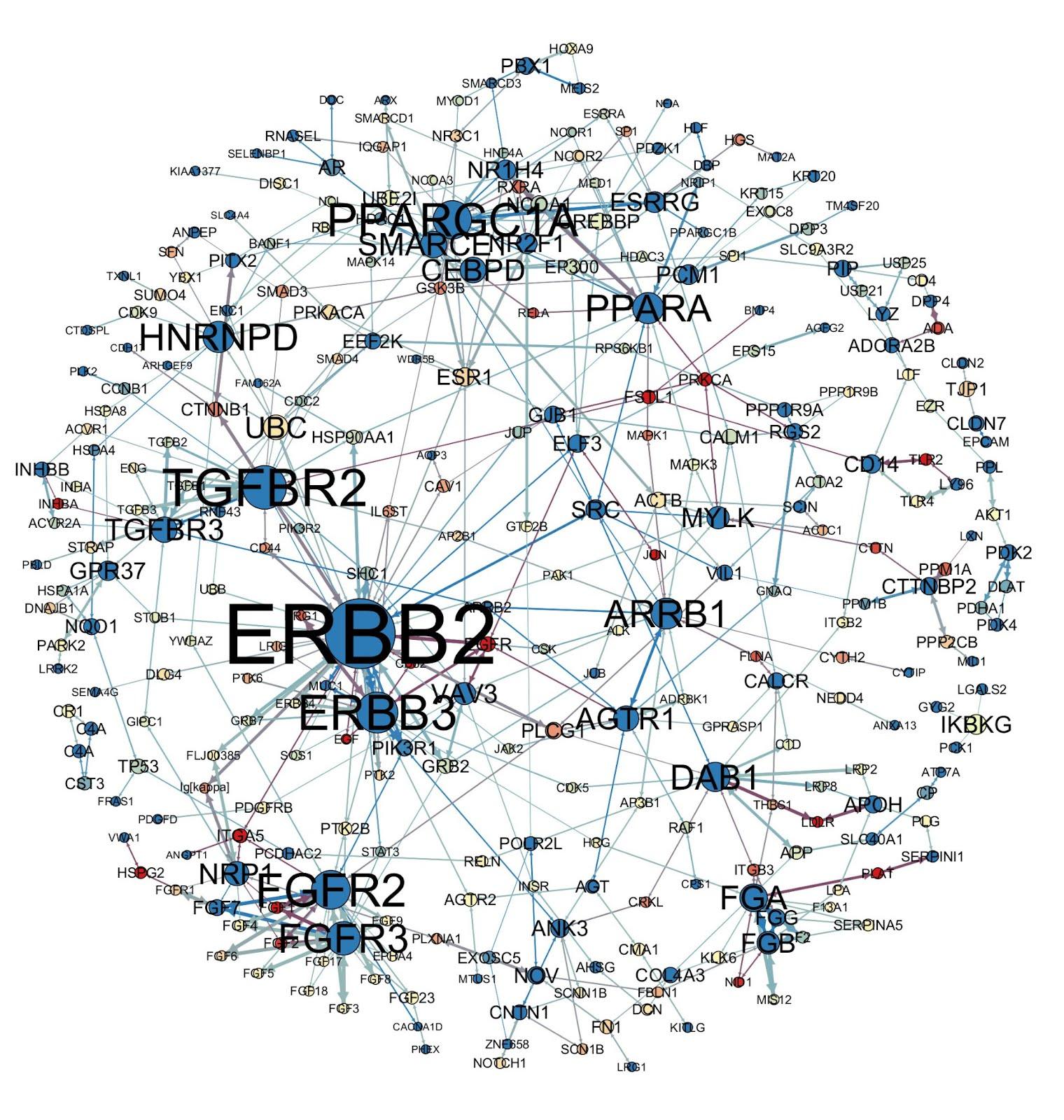


Protein-protein interaction network induced by the most down-regulated genes. This network contains genes that are 4-fold or more down-regulated and genes that mediate interactions between down-regulated genes. Color of nodes corresponds to differential gene expression (blue – down, red – up, white – no change). Size of nodes corresponds to the PageRank.
